# Supplementary material for: Electrolyte design principles for developing quasi-solid-state rechargeable halide-ion batteries
Source: Nat Commun. 2023 Feb 18;14:925. doi: 10.1038/s41467-023-36622-w (PMC9938900; doi:10.1038/s41467-023-36622-w)
Supplement: Supplementary file 1 — Supplementary Information [file 41467_2023_36622_MOESM1_ESM.docx]

**Supplementary Information**

**Electrolyte design principles for developing quasi–solid–state rechargeable halide–ion batteries**

*Xu Yang^1 ,2^, Bao Zhang^3,4^, Yao Tian^2^, Yao Wang^2^, Zhiqiang Fu^2^, Dong Zhou^2, *^, Hao Liu^1^, Feiyu Kang^2^, Baohua Li^2, *^, Chunsheng Wang^3, *^, Guoxiu Wang^1, *^*

^1^ Centre for Clean Energy Technology, School of Mathematical and Physical Sciences, Faculty of Science, University of Technology Sydney, Sydney, NSW 2007, Australia

^2^ Tsinghua Shenzhen International Graduate School, Tsinghua University, Shenzhen 518055, China

^3^ Department of Chemical and Biomolecular Engineering, University of Maryland, College Park, Maryland 20742, United States

^4^ School of Optical and Electronic Information, Huazhong University of Science and Technology, Wuhan, 430074, P. R. China

* Email: zhou.d@sz.tsinghua.edu.cn

libh@sz.tsinghua.edu.cn

cswang@umd.edu

Guoxiu.Wang@uts.edu.au


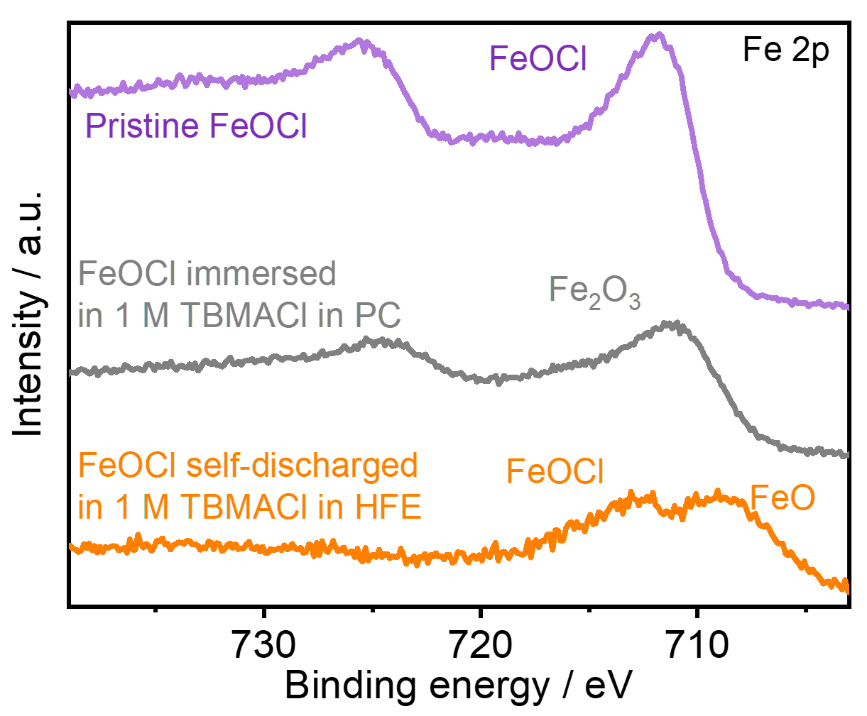


**Supplementary Figure 1** Fe 2p XPS spectra of pristine FeOCl, FeOCl cathode after immersed in 1 M TBMACl in PC electrolyte for 7 days, and FeOCl cathode self–discharged in a Li|1 M TBMACl in HFE|FeOCl cell for 100 h. The corresponding Cl/Fe atomic ratio obtained from XPS data is listed in **Supplementary Table 4**. It is seen that the peak at 712.1 eV in the pristine FeOCl spectrum is assigned to FeOCl^1^. After immersed in PC, a new peak at around 711.1 eV appears, corresponding to the formation of Fe_2_O_3_ based on **Equation 3**^2^. Meanwhile, the FeOCl cathode self–discharged in a Li|1 M TBMACl in HFE|FeOCl cell exhibited a FeO peak at about 708.9 eV,^1^ which is well consistent with **Equation 4** (“Equation 3” and “Equation 4” are in the main text of the manuscript).


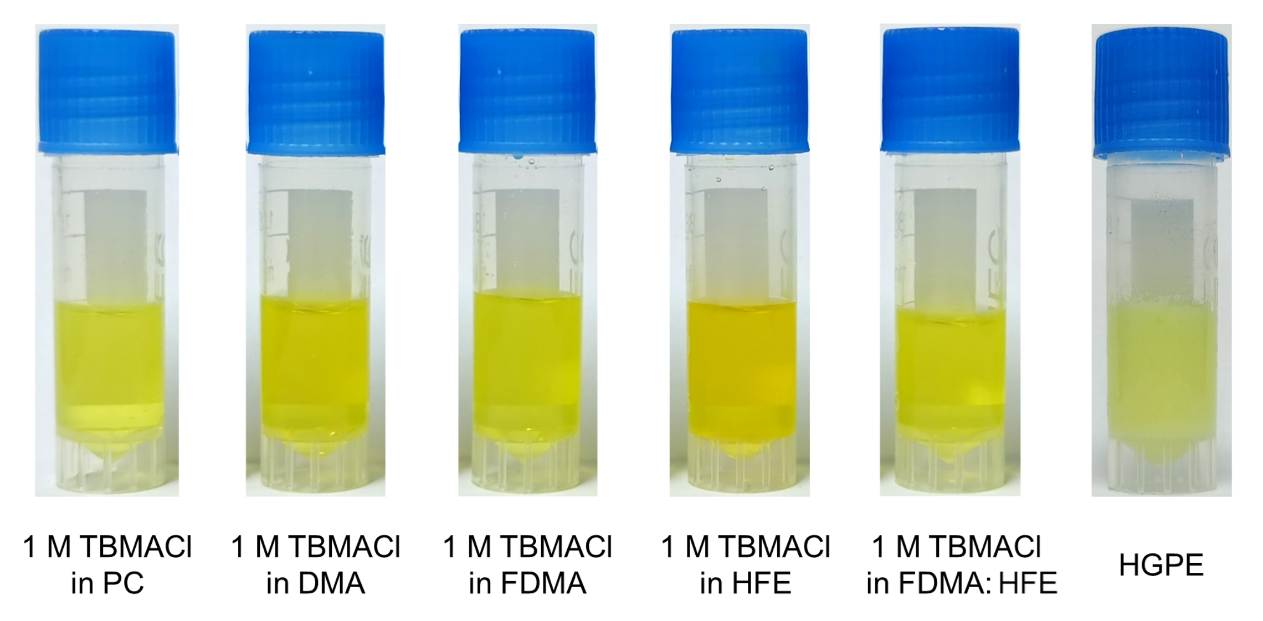


**Supplementary Figure 2** Optical images of saturated Cl_2_ dissolved in 1 M TBMACl in PC, 1 M TBMACl in DMA, 1 M TBMACl in FDMA, 1 M TBMACl in HFE, 1 M TBMACl in FDMA: HFE electrolytes and HGPE, respectively.


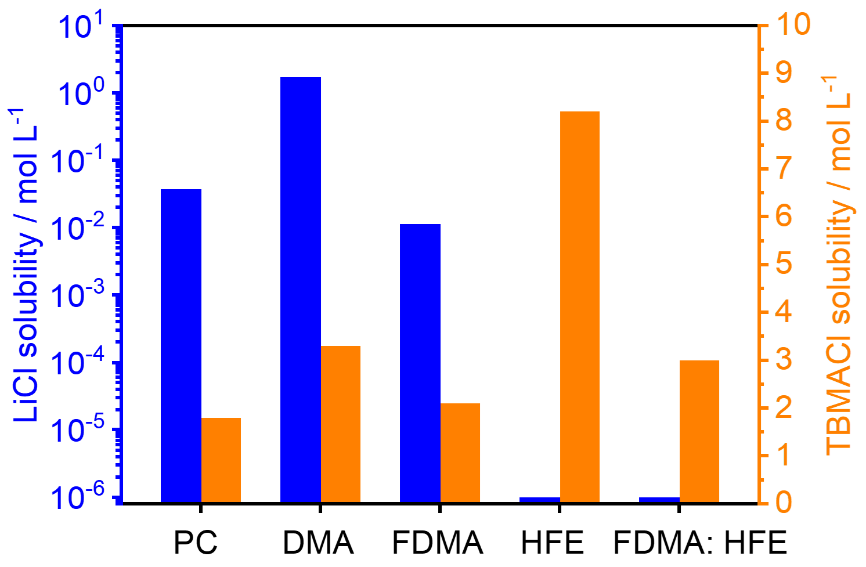


**Supplementary Figure 3** LiCl and TBMACl solubility in PC, DMA, FDMA, HFE, and FDMA: HFE (1: 1 by volume) solvents at 25 °C, respectively.

**
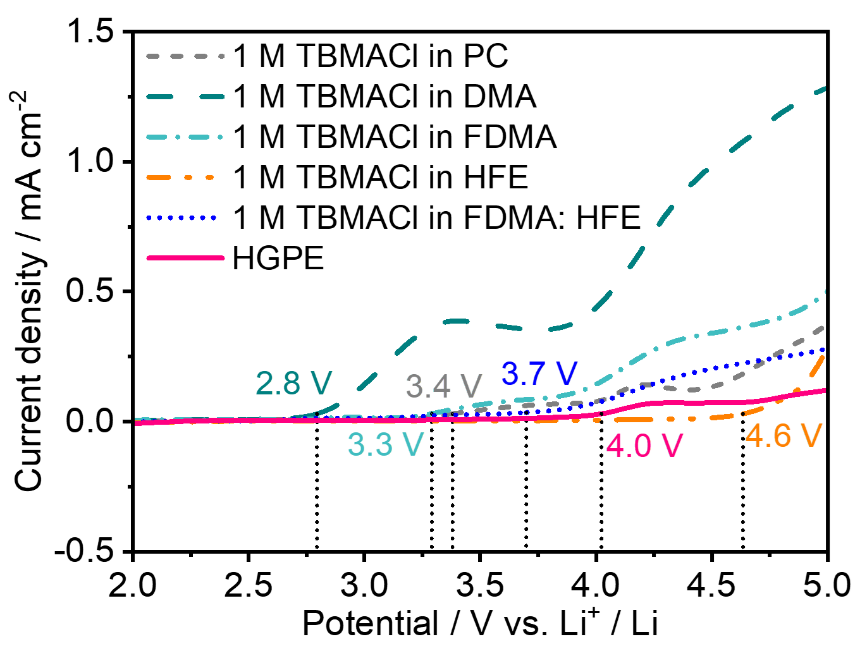
**

**Supplementary Figure 4** LSV curves of different electrolytes at 25 °C and a scan rate of 10 mV s^–1^ using titanium as the working electrode and Li as the counter and reference electrodes in coin cell.


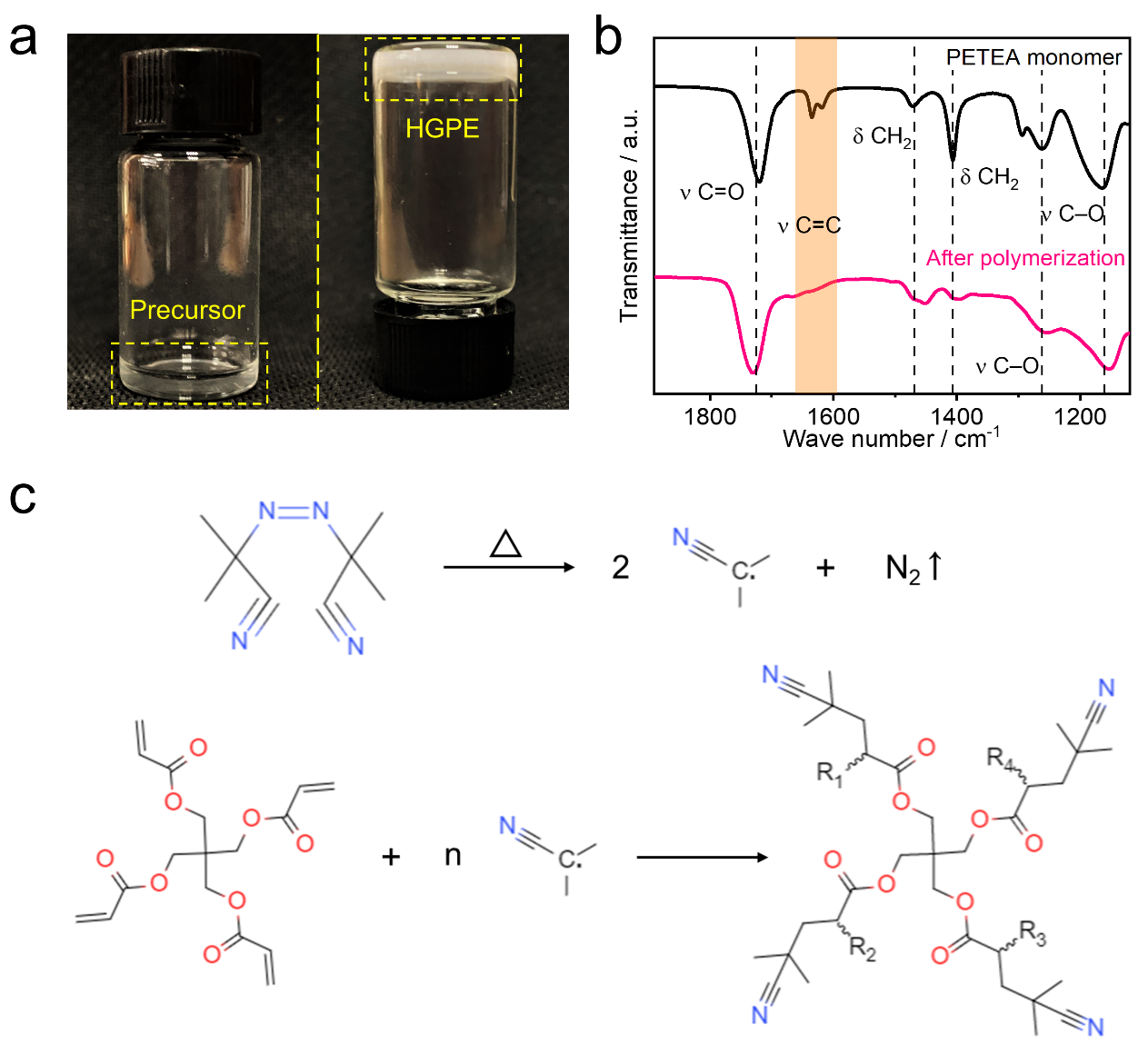


**Supplementary Figure 5** **a** Optical images of precursor solution (left) and HGPE (right). **b** FTIR spectra of PETEA monomer and the polymer matrix of HGPE. **c** Polymerization mechanism of the PETEA monomers^3^. R_1_, R_2_, R_3,_ and R_4_ represent molecular chains (see **Supplementary Note 1**).


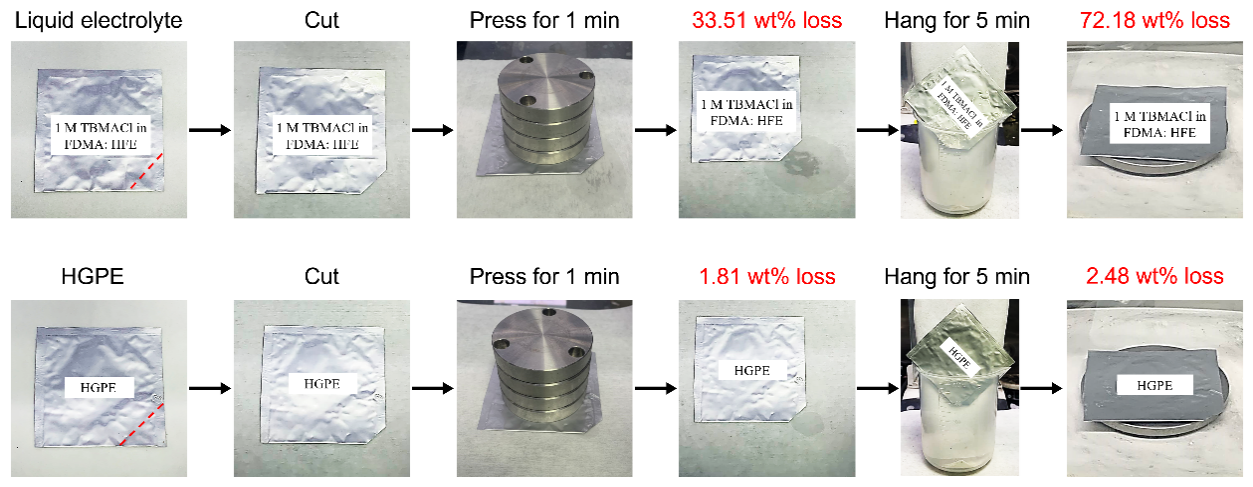


**Supplementary Figure 6** Leakage tests of 1 M TBMACl in FDMA: HFE liquid electrolyte (upper panels) and HGPE (lower panels) (see **Supplementary Note 2**).


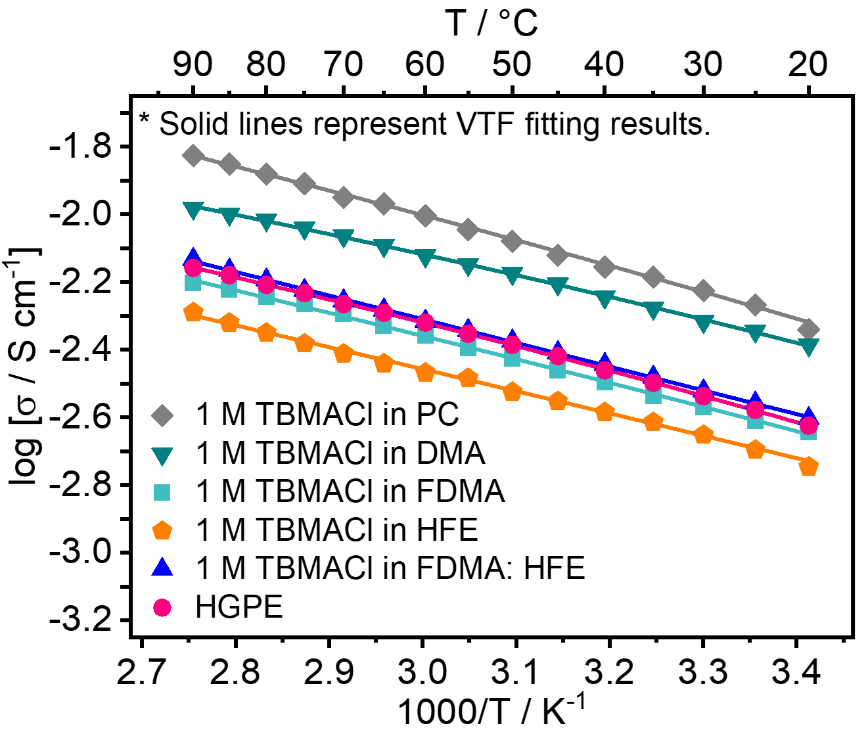


**Supplementary Figure 7** Bulk ionic conductivity (σ) values of different liquid electrolytes and HGPE in a temperature range of 20–90 °C. Dots represent the experimental data while solid lines represent Vogel–Tammann–Fulcher (VTF) fitting results (**Supplementary Table 5**).^4^


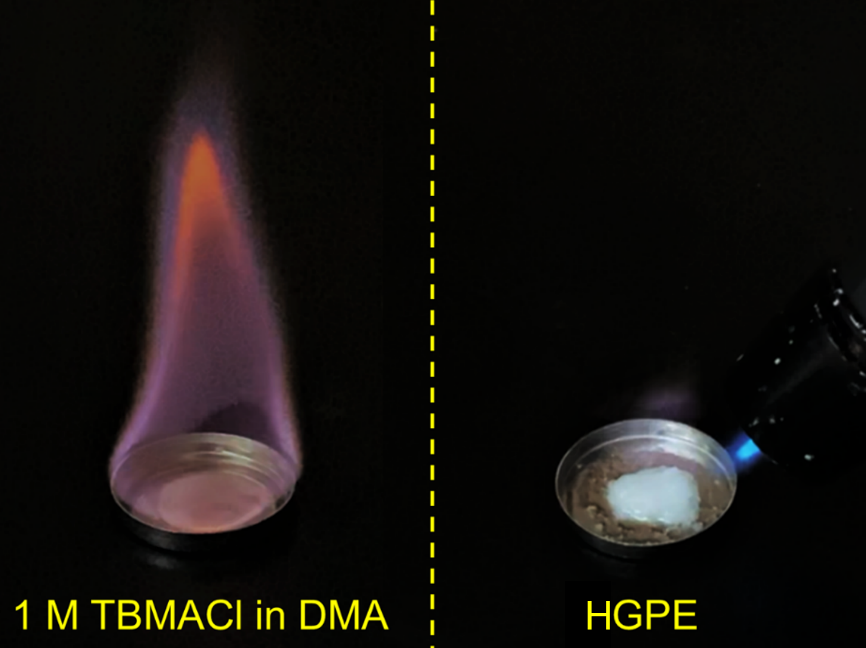


**Supplementary Figure 8** Optical images of 1 M TBMACl in DMA electrolyte and HGPE after ignited. During the combustion test, 1 M TBMACl in the DMA electrolyte was ignited with a self–extinguishing time (SET) of 50 s g^–1^ (left panel and **Supplementary Movie 1**), while the HGPE exhibited non–flammability with a SET of 0 s g^–1^ (right panel and **Supplementary Movie 2**).


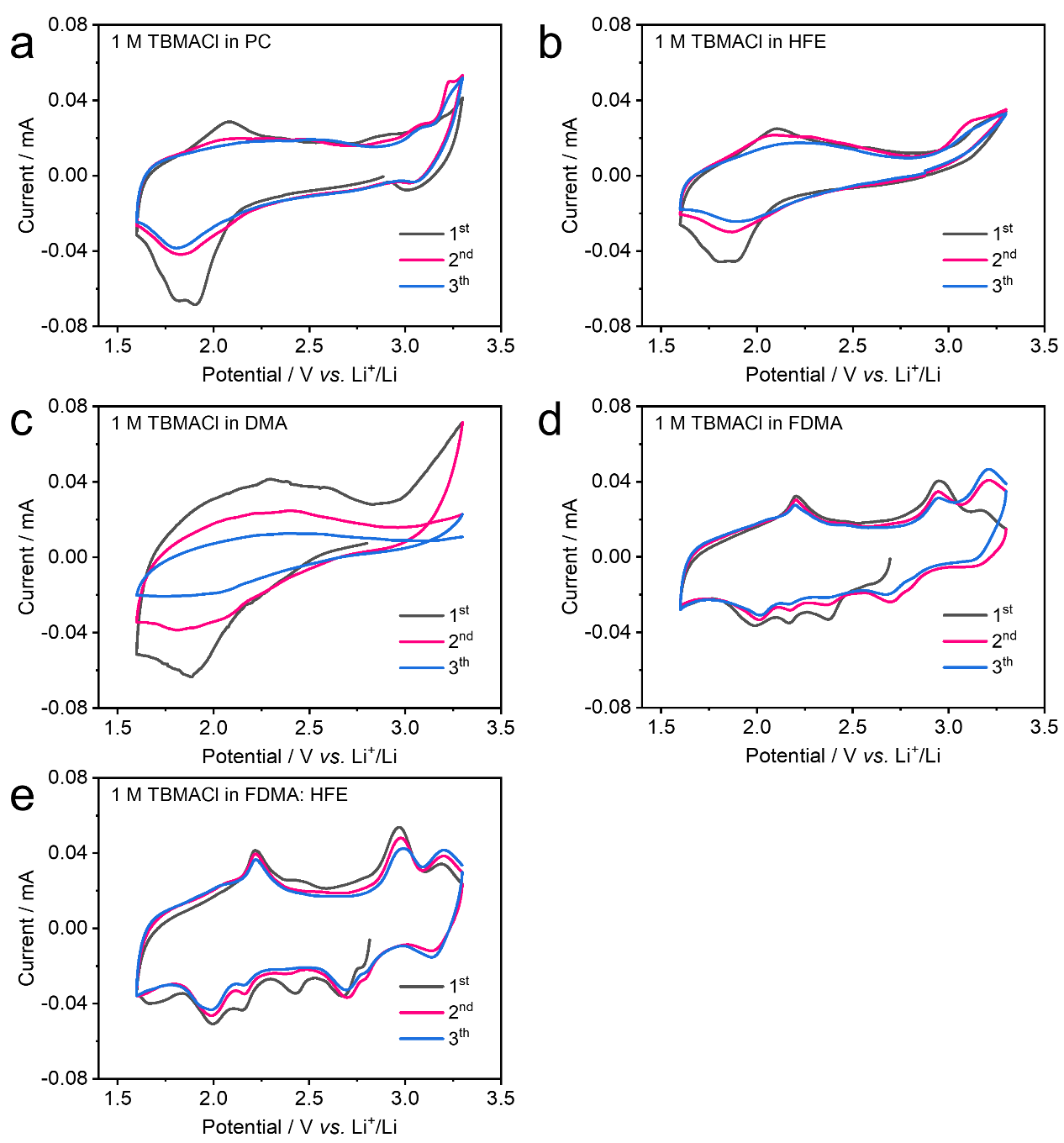


**Supplementary Figure 9** CV curves of Li||FeOCl coin cells at 25 °C with **a** 1 M TBMACl in PC, **b** 1 M TBMACl in HFE, **c** 1 M TBMACl in DMA, **d** 1 M TBMACl in FDMA, and **e** 1 M TBMACl in FDMA: HFE electrolytes at a scan rate of 0.1 mV s^–1^.

**
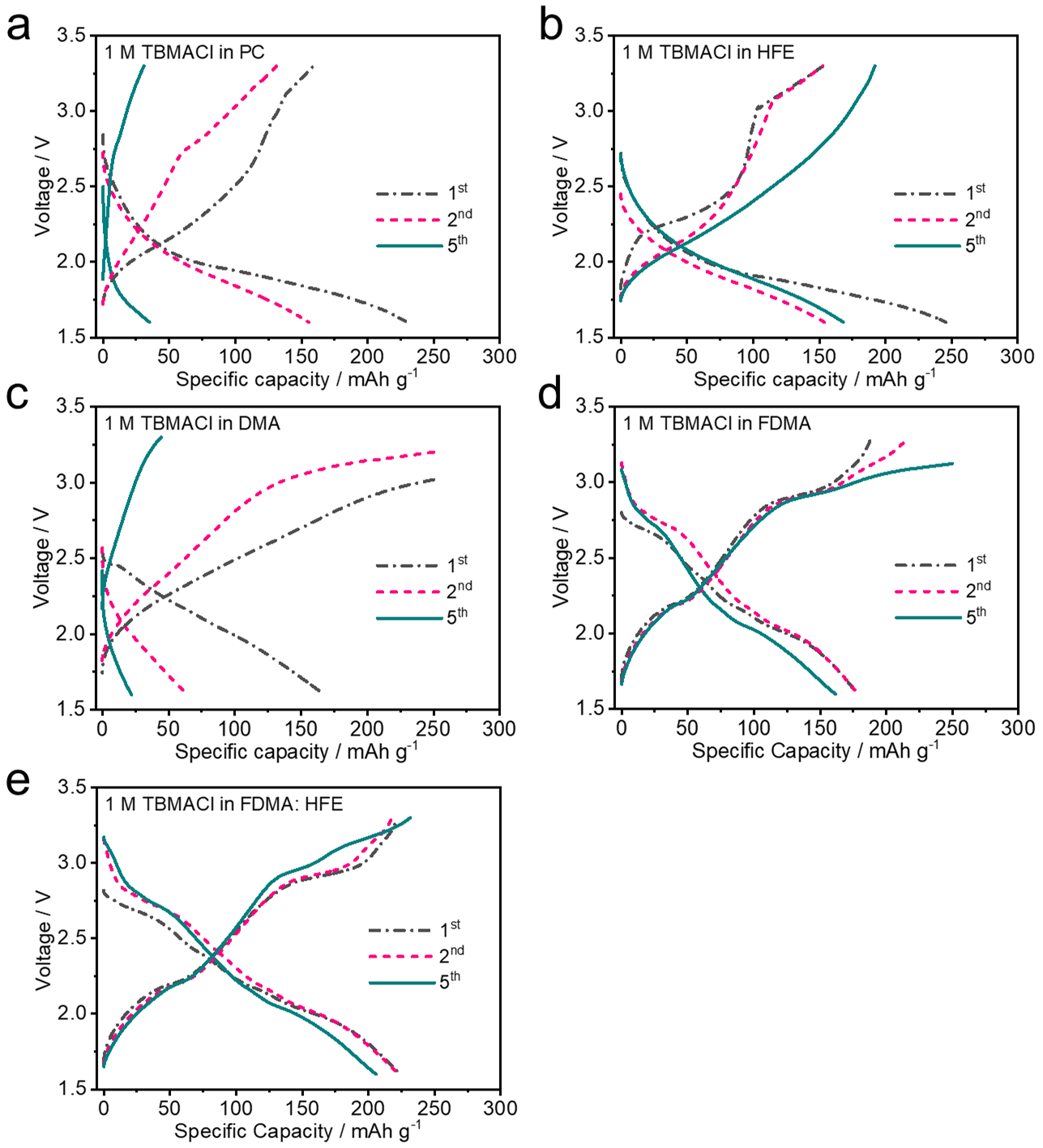
**

**Supplementary Figure 10** Typical discharge/charge profiles of the Li||FeOCl cells with **a** 1 M TBMACl in PC, **b** 1 M TBMACl in HFE, **c** 1 M TBMACl in DMA, **d** 1 M TBMACl in FDMA, and **e** 1 M TBMACl in FDMA: HFE electrolytes at 125 mA g^–1^ and 25 °C.


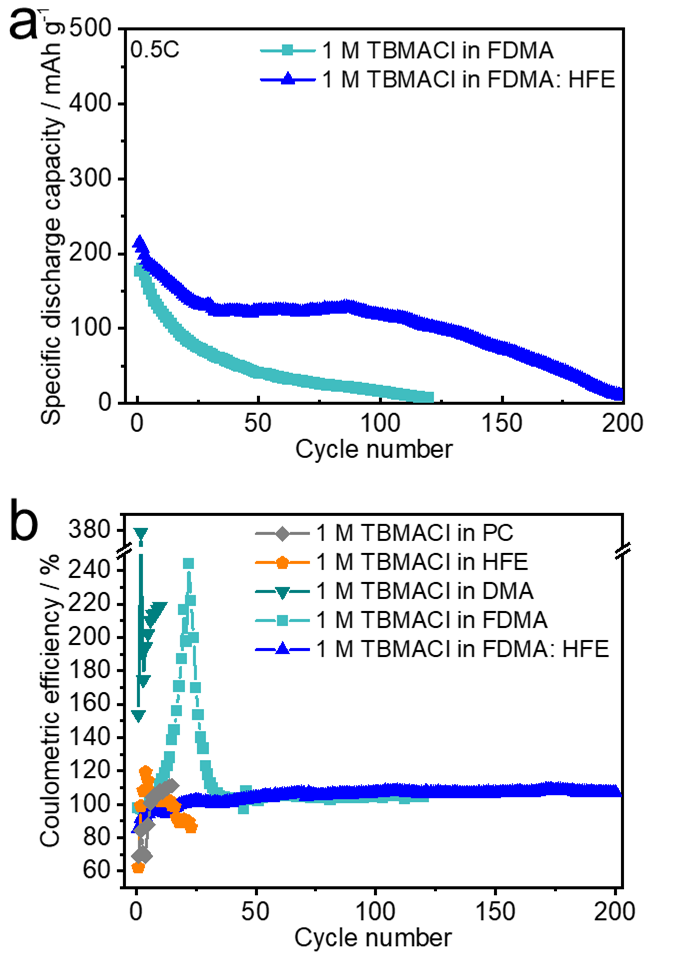


**Supplementary Figure 11 a** Cycling performance of the Li||FeOCl cells with 1 M TBMACl in FDMA and 1 M TBMACl in FDMA: HFE electrolytes at 125 mA g^–1^ and 25 °C. **b** Coulombic efficiency of the Li||FeOCl cells with different electrolytes at 125 mA g^–1^ and 25 °C.


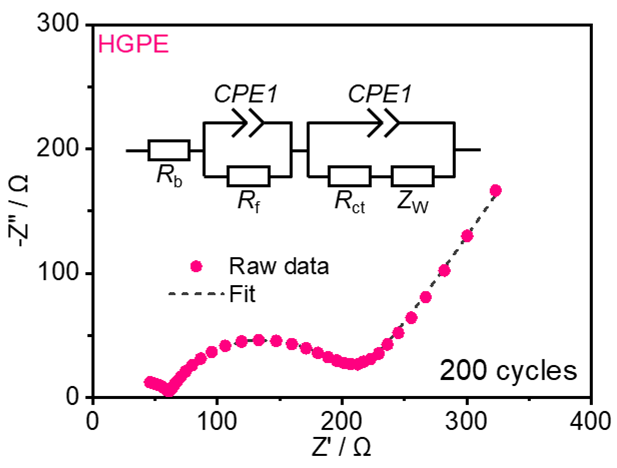


**Supplementary Figure 1****2** The raw and the fitted data of EIS curves of the Li|HGPE|FeOCl cell after 500 cycles at 125 mA g^–1^ and 25 °C using an equivalent circuit^5^ shown in the inset (see **Supplementary Note 3**). The numerical values for each equivalent circuit element and the error between the raw and fitted data are listed in **Supplementary Table 6**.


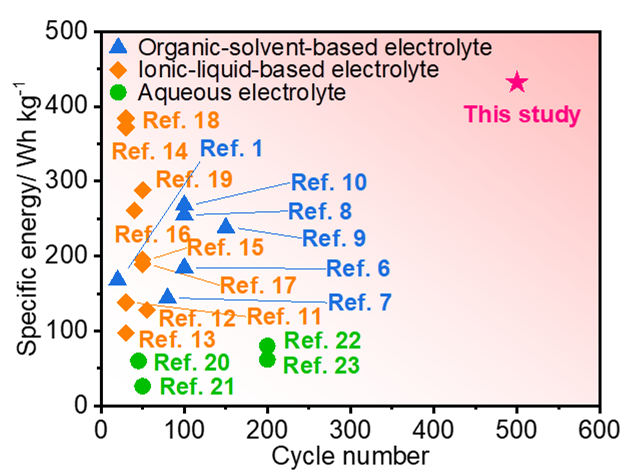


**Supplementary Figure 13** Comparison of specific energy (based on the mass of cathode active material only) and cycle life of reported CIBs using organic–solvent–based^1, 6–10^ ionic–liquid–based^11–19^ or aqueous^20–23^ electrolytes and the quasi–solid–state CIB in this work. The data are listed in the **Supplementary Table 7**.


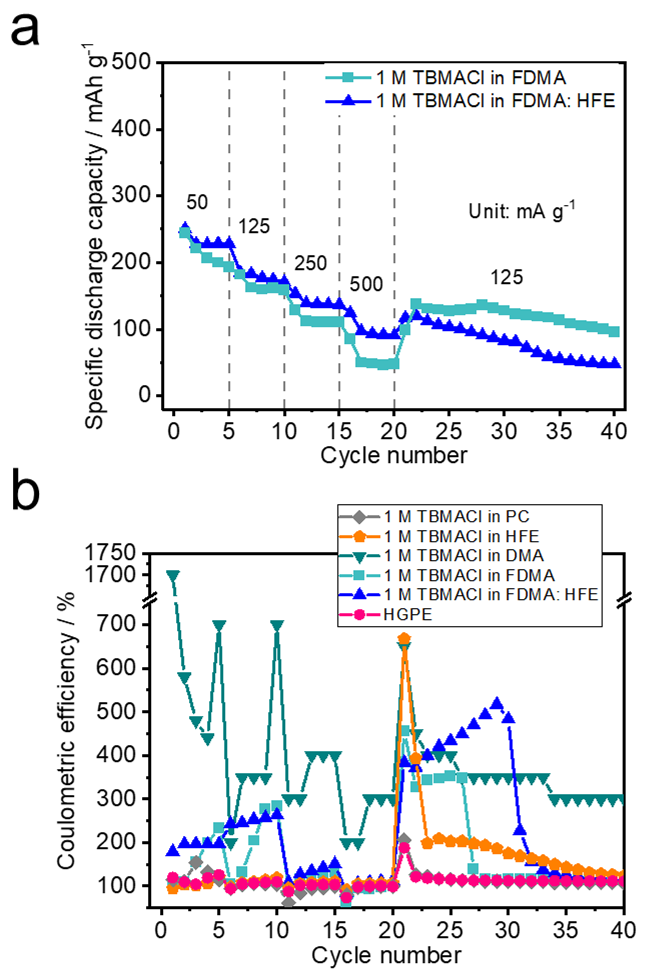


**Supplementary Figure 14 a** Rate performance of the Li||FeOCl coin cells with 1 M TBMACl in FDMA and 1 M TBMACl in FDMA: HFE electrolytes at 125 mA g^–1^ and 25 °C. **b** Coulombic efficiencies of the rate performance of Li||FeOCl cells with different electrolytes at 125 mA g^–1^ and 25 °C.


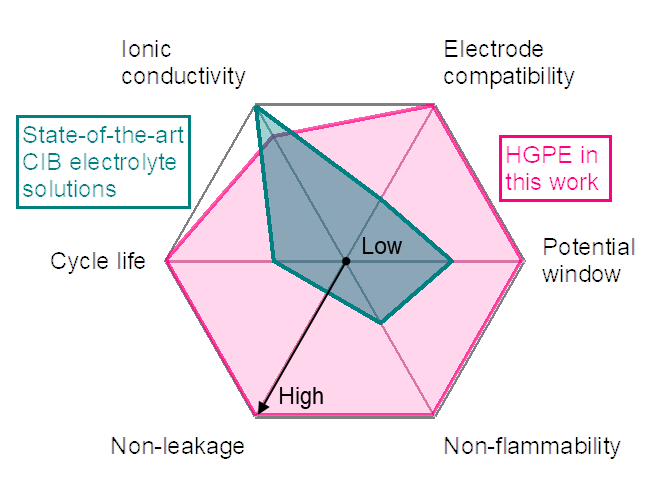


**Supplementary Figure 15** Performance comparison of electrolytes for conventional CIBs and HGPE in this work.


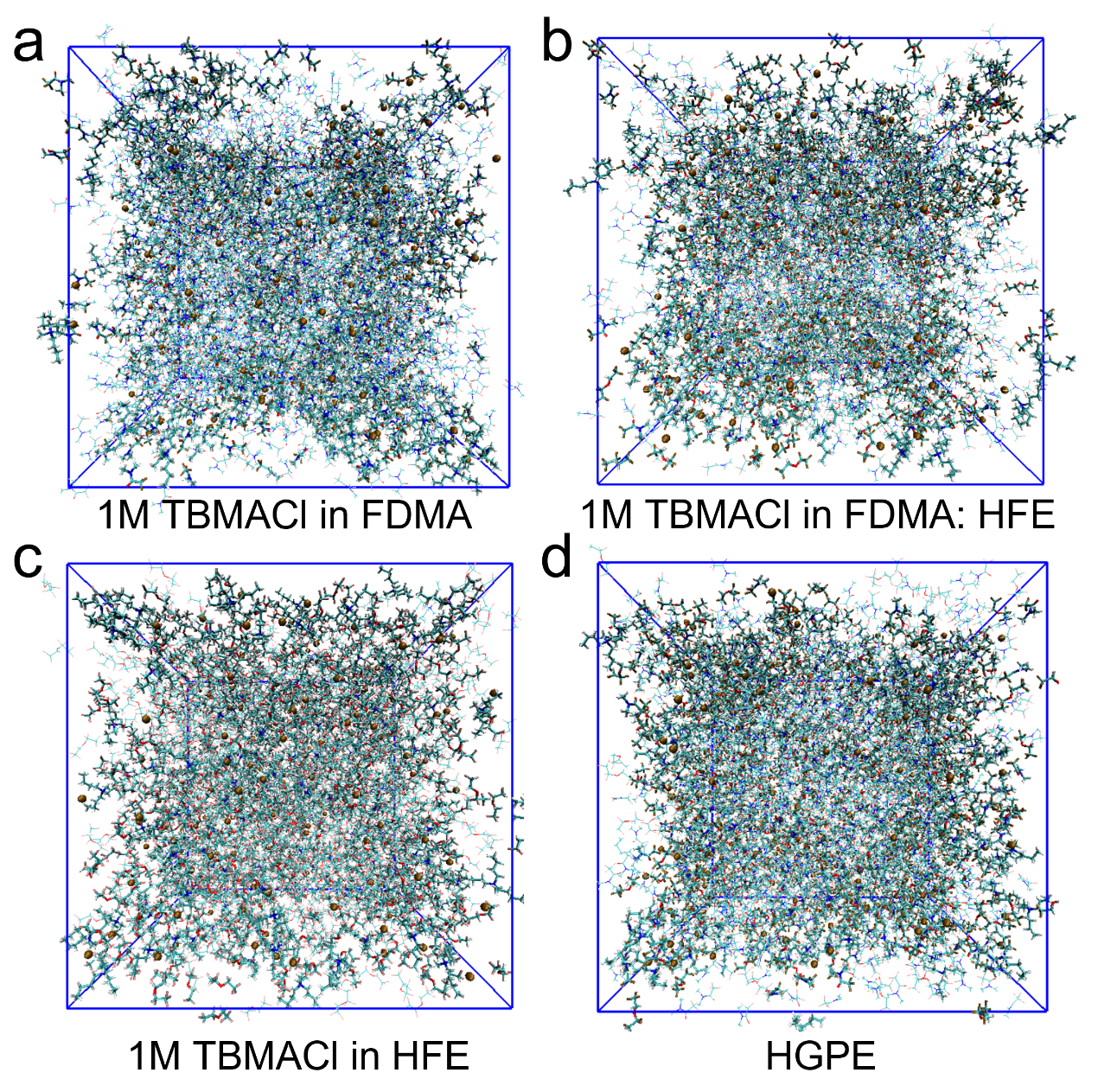


**Supplementary Figure 16 a–d** Snapshots obtained from MD simulations of **a** 1 M TBMACl in FDMA, **b** 1 M TBMACl in FDMA: HFE, and **c** 1 M TBMACl in HFE electrolytes, and **d** HGPE.


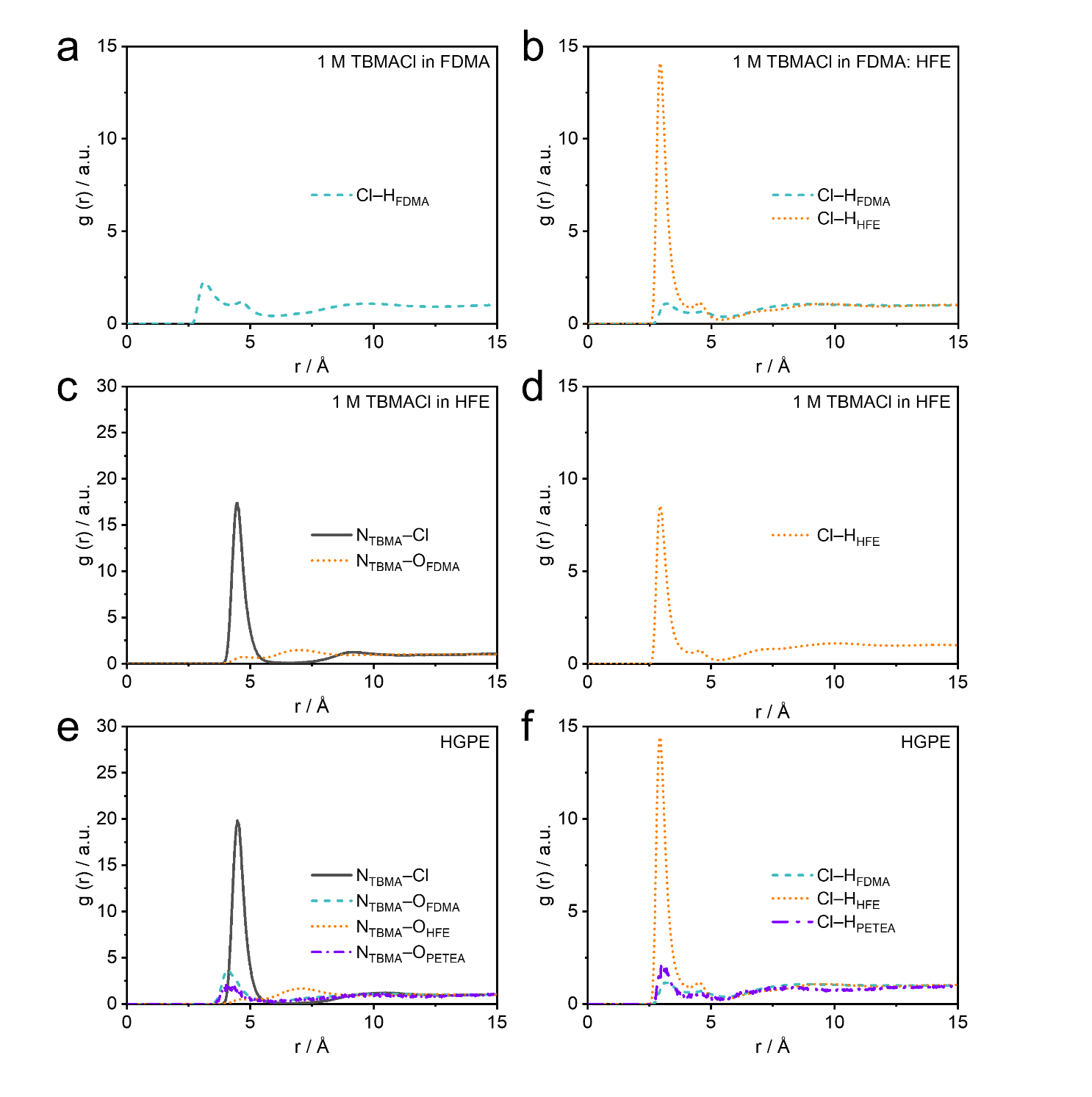


**Supplementary Figure 17 a–f** RDFs obtained from MD simulations. **a, b** RDFs of Cl^–^–coordination pairs for **a** 1 M TBMACl in FDMA and ­**b** 1 M TBMACl in FDMA: HFE electrolytes. **c–f** RDFs of **c**, **e** TBMA^+^–coordination, and **d**, **f** Cl^–^–coordination pairs for **c, d** 1 M TBMACl in HFE electrolyte and **e, f** HGPE.


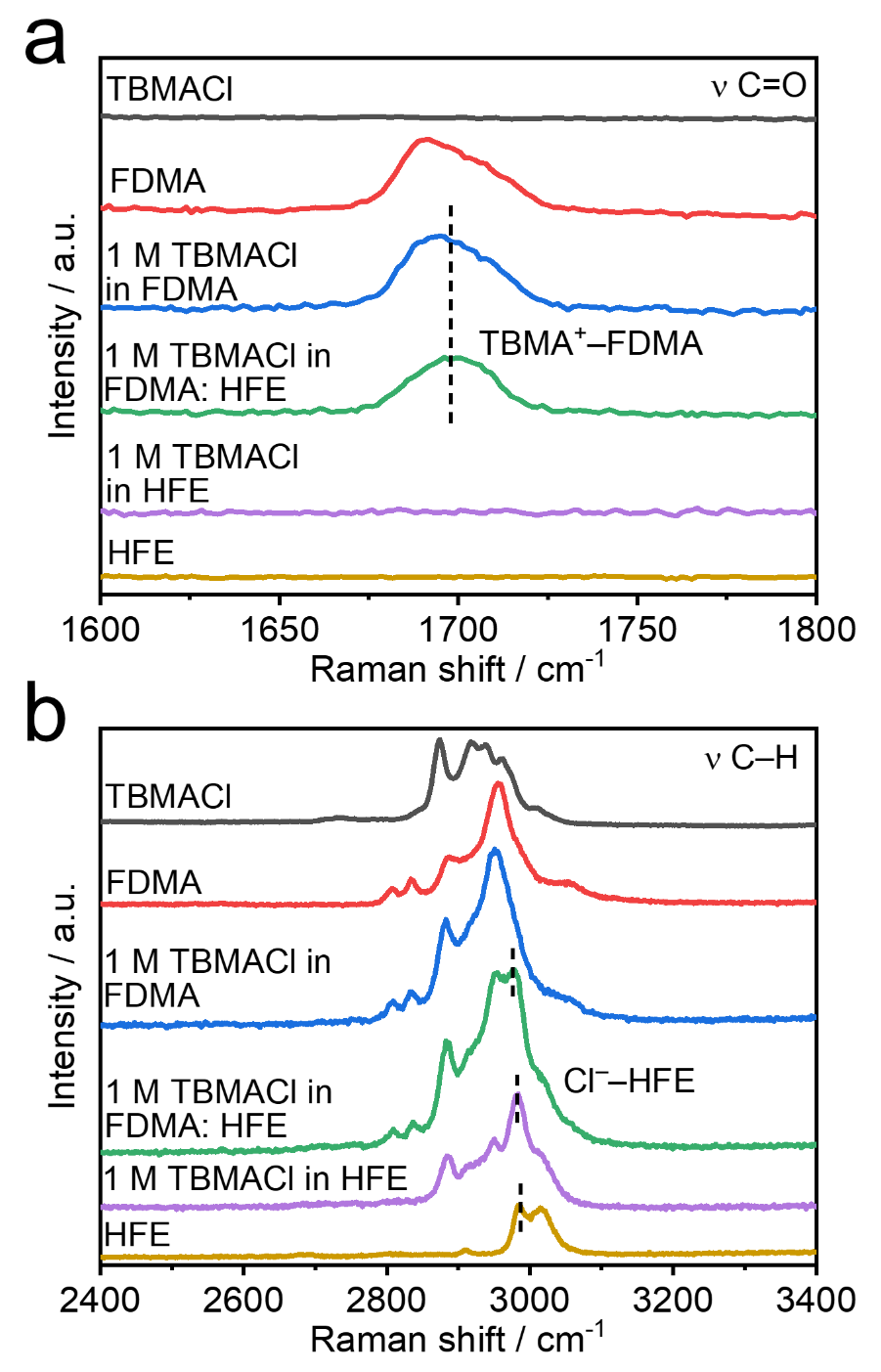


**Supplementary Figure 18 a, b** Raman spectra of TBMACl salt, FDMA and HFE solvents, and different electrolytes at 25 °C.


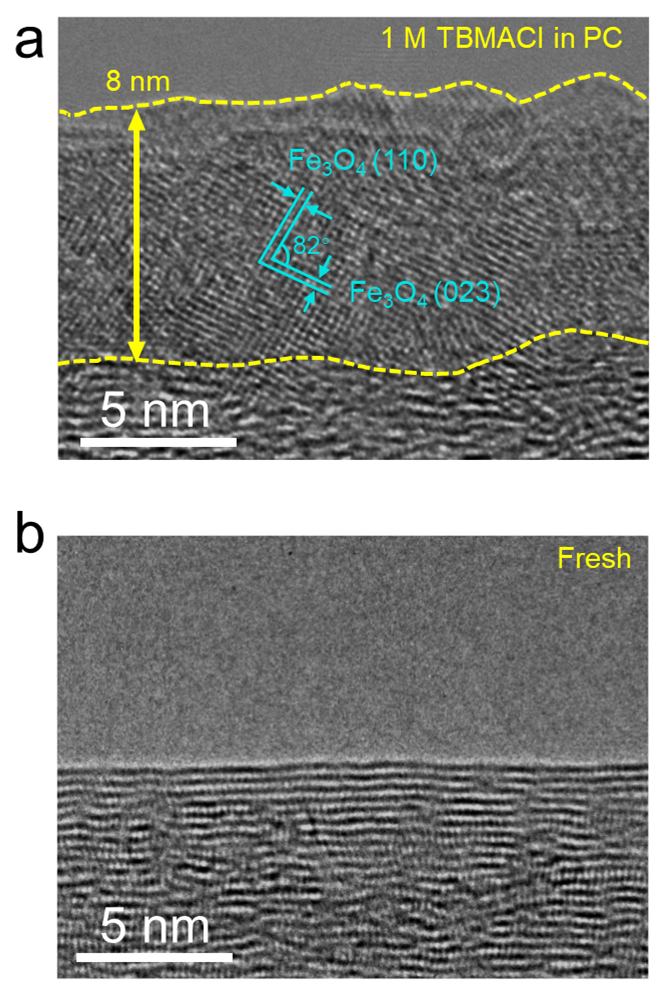


**Supplementary Figure 19** TEM images of **a** fully charged FeOCl particles obtained from Li||FeOCl coin cells with 1 M TBMACl in PC electrolytes after 3 cycles at 125 mA g^–1^ and 25 °C and **b** pristine FeOCl particles. The crystalline phases with interplanar spacings of 0.268 and 0.262 nm as well as a cross angle of 82° are assigned to the (110) and (023) plane of Fe_3_O_4_ (PDF#01–089–6466), respectively.


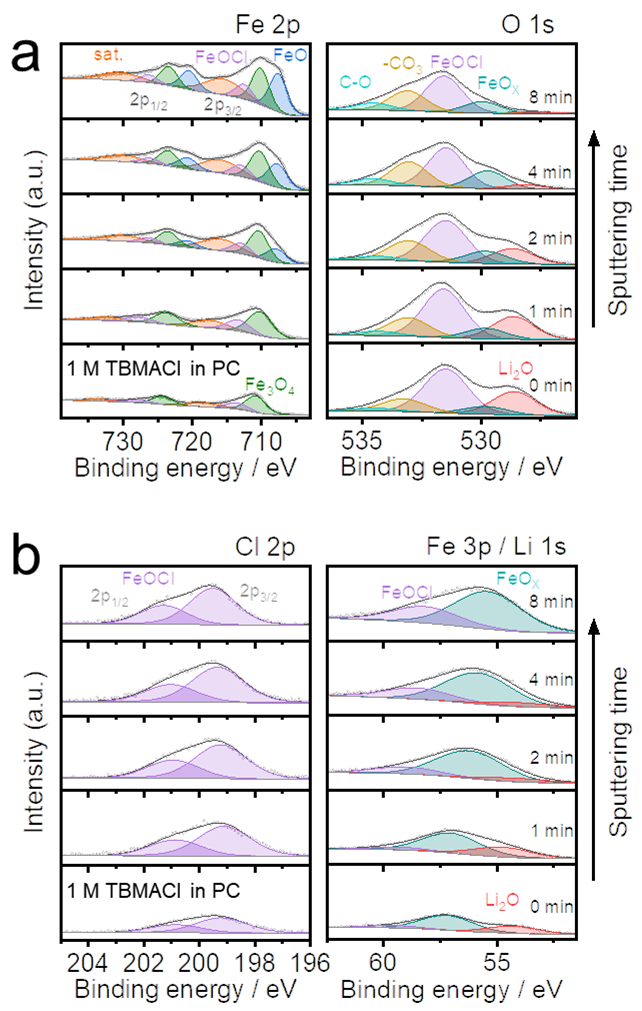


**Supplementary Figure 20** *Ex situ* in–depth XPS spectra of fully charged FeOCl–based positive electrodes obtained from Li|1 M TBMACl in PC|FeOCl coin cells after 3 cycles at 125 mA g^–1^ and 25 °C. **a** Fe 2p, O 1s and **b** Cl 2p, Fe 3p/Li 1s XPS spectra (see **Supplementary Note 5**).


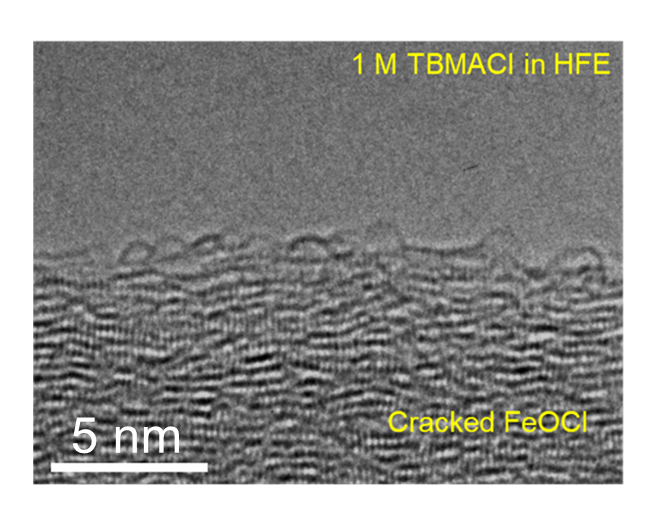


**Supplementary Figure 21** TEM images of **a** fully charged FeOCl particles obtained from Li||FeOCl coin cells with 1 M TBMACl in PC electrolytes after 3 cycles at 125 mA g^–1^ and 25 °C.


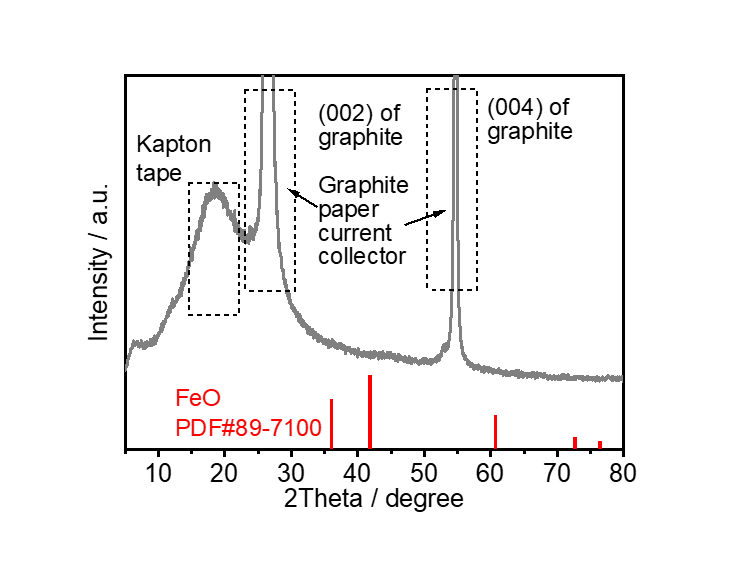


**Supplementary Figure 22** XRD patterns of the fully–discharged FeOCl cathode protected by a Kapton tape. To avoid the oxidation of FeO by air during sample preparation^14^, the FeOCl electrode from disassembled the Li||FeOCl cell was tested by XRD the under the protection of a Kapton tape. No characteristic peaks of FeO were found in the XRD pattern, indicating the amorphous structure of FeO as discharge product.


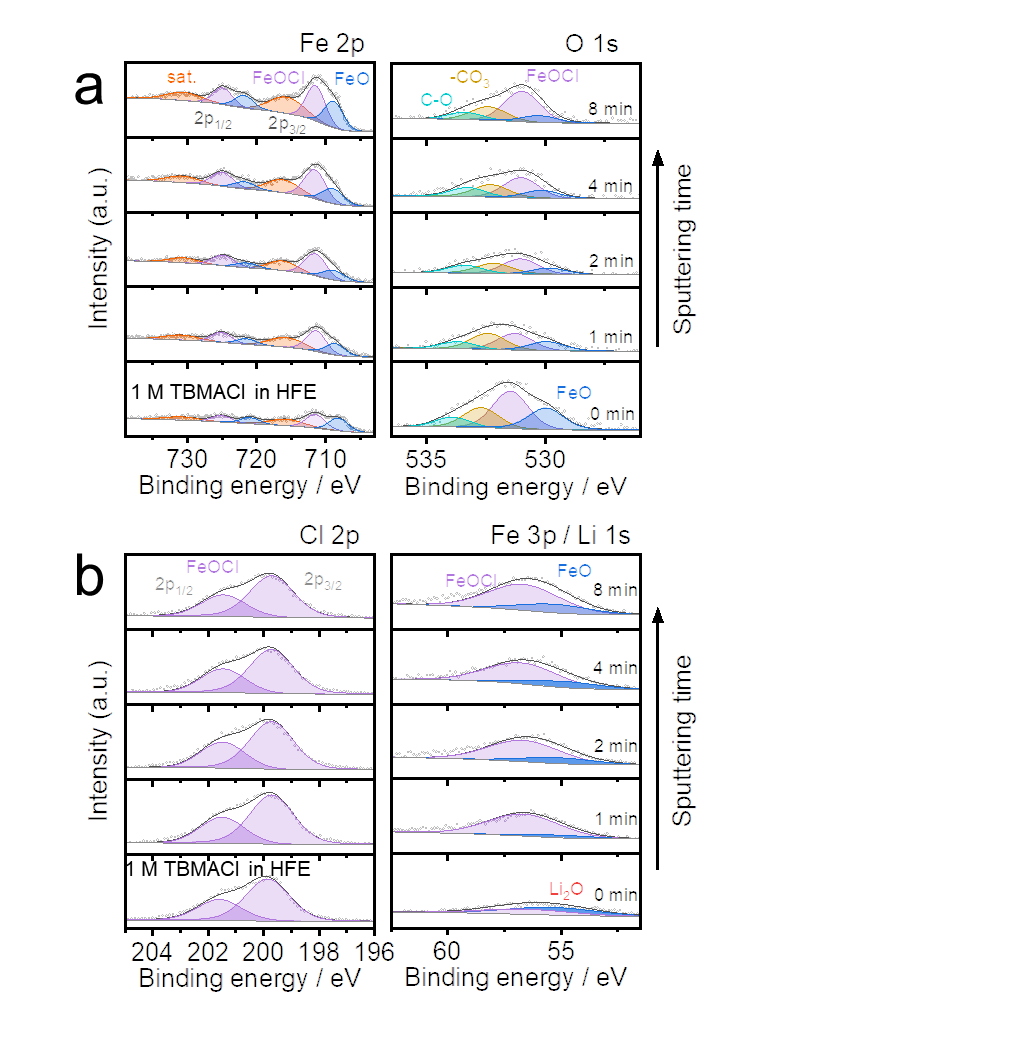


**Supplementary Figure 23** *Ex situ* in–depth XPS spectra of fully charged FeOCl–based positive electrodes obtained from Li|1 M TBMACl in HFE|FeOCl coin cells after 3 cycles at 125 mA g^–1^ and 25 °C. **a** Fe 2*p*, O 1s and **b** Cl 2*p*, Fe 3*p*/Li 1s XPS spectra (see **Supplementary Note 5**).


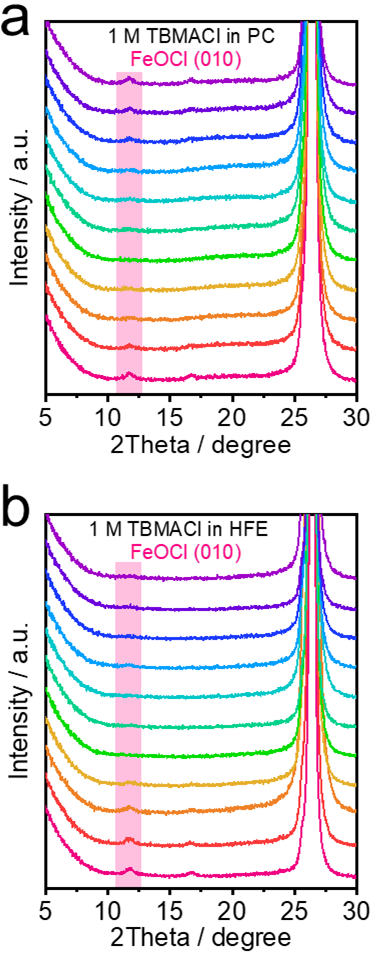


**Supplementary Figure 24** Typical galvanostatic discharge/charge profiles at 50 mA g^–1^ and 25 °C and corresponding *ex situ* XRD patterns of Li||FeOCl cells with **a** 1 M TBMACl in PC electrolyte and **b** HGPE.


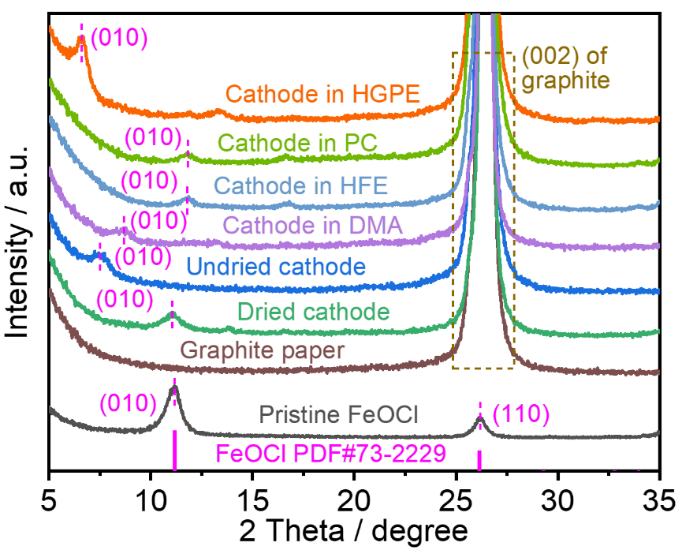


**Supplementary Figure 25** XRD patterns of pristine FeOCl particles, graphite paper as the current collector, undried FeOCl cathode, dried FeOCl cathode, and FeOCl cathode after immersed in 1 M TBMACl in PC, HFE, DMA electrolyte, and HGPE (see **Supplementary Note 6**).


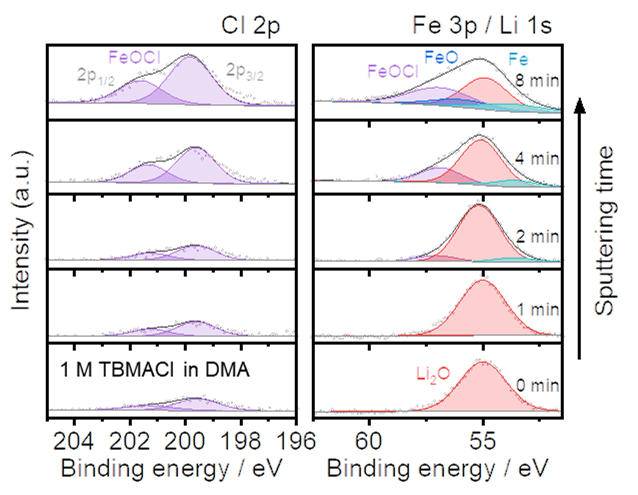


**Supplementary Figure 26** *Ex situ* in–depth Cl 2*p*, Fe 3p/Li 1*s* XPS spectra of fully charged FeOCl–based positive electrodes obtained from Li|1 M TBMACl in DMA|FeOCl coin cells after 3 cycles at 125 mA g^–1^ and 25 °C (see **Supplementary Note 5**).


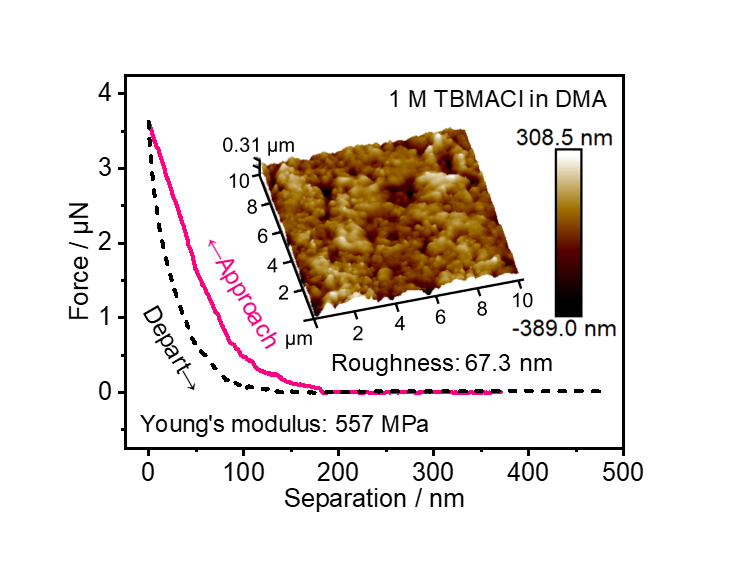


**Supplementary Figure 27** Force–displacement plots of the FeOCl surface cycled in 1 M TBMACl in DMA electrolytes at 125 mA g^–1^ and 25 °C. The corresponding 3D–AFM scanning images of cathodes are shown in the insets.


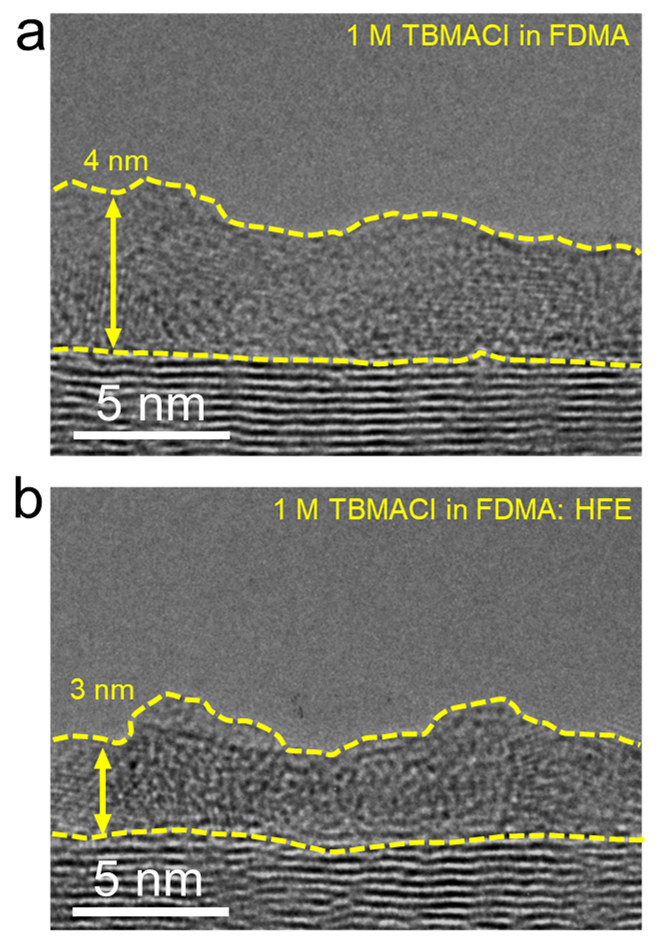


**Supplementary Figure 28** TEM images of fully charged FeOCl particles obtained from Li||FeOCl coin cells with **a** 1 M TBMACl in FDMA, and **b** 1 M TBMACl in FDMA: HFE electrolytes after 3 cycles at 125 mA g^–1^ and 25 °C.


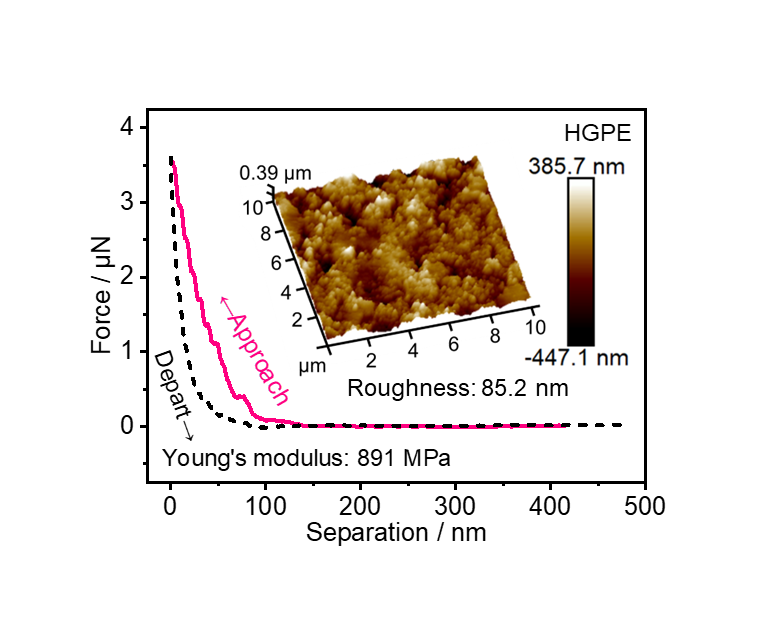


**Supplementary Figure 29** Force–displacement plots of the FeOCl surface cycled in HGPE at 125 mA g^–1^ and 25 °C. The corresponding 3D–AFM scanning images of cathodes are shown in the insets.


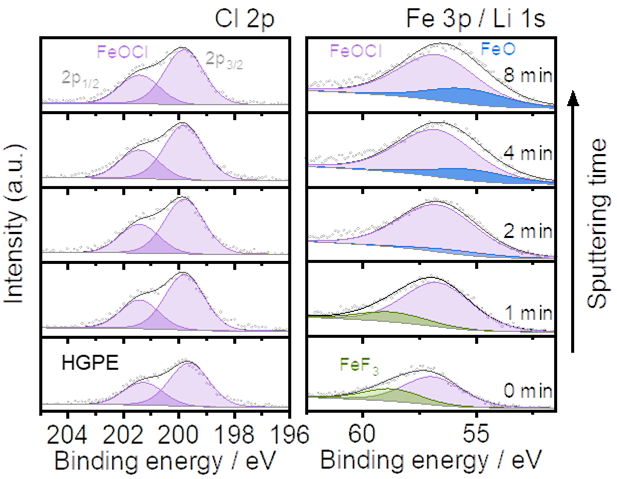


**Supplementary Figure 30** *Ex situ* in–depth Cl 2*p*, Fe 3*p*/Li 1*s* XPS spectra of fully charged FeOCl–based positive electrodes obtained from Li|HGPE|FeOCl coin cells after 3 cycles at 125 mA g^–1^ and 25 °C (see **Supplementary Note 5**).


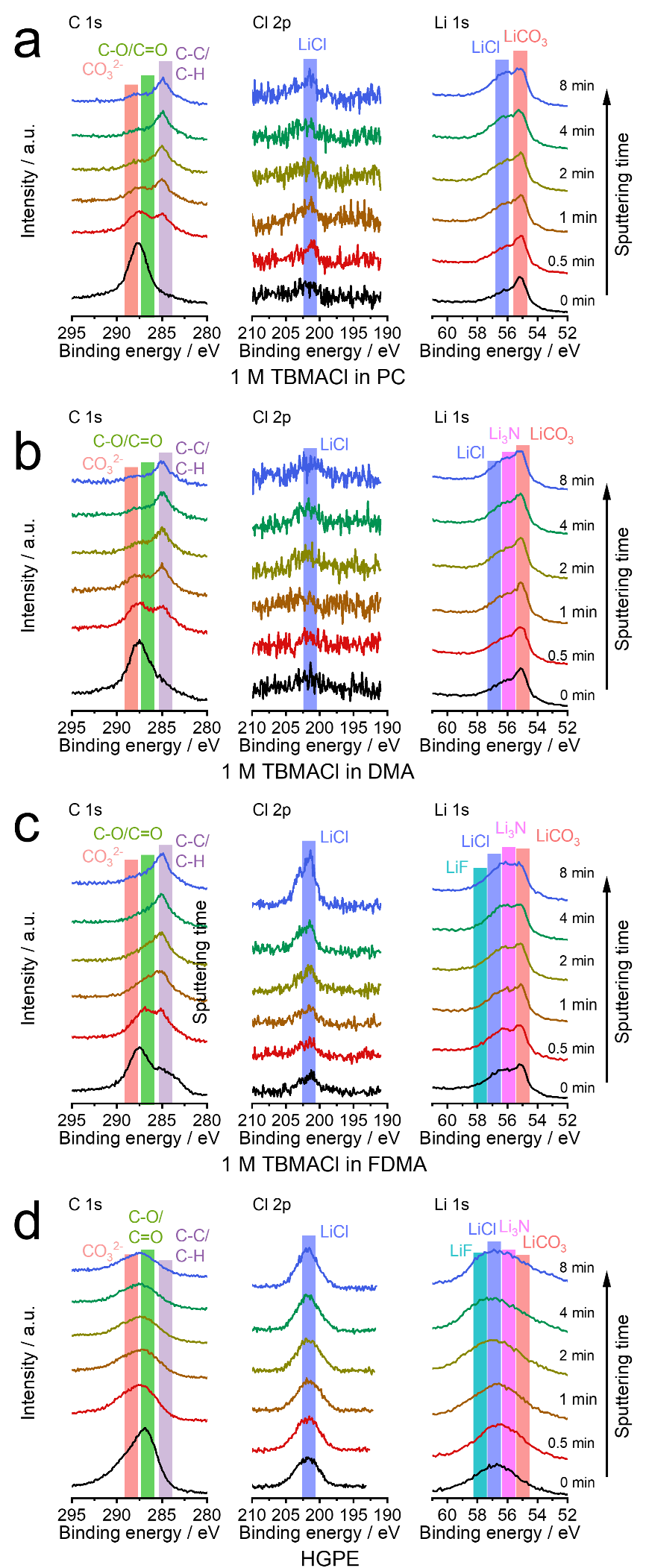


**Supplementary Figure 31** In–depth XPS spectra of fully discharged Li metal anodes for 3 cycles obtained from Li||FeOCl coin cells at 125 mA g^–1^ and 25 °C with **a** 1 M TBMACl in PC, **b** 1 M TBMACl in DMA, **c** 1 M TBMACl in FDMA electrolytes, and **d** HGPE. C 1*s*: CO_3_^2–^: 288.5 eV; C–O/C=O: 286.6 eV; C–C/C–H: 284.8 eV^24^. Li 1*s*: Li_2_CO_3_: 55.0 eV; Li_3_N: 56.0 eV^25^; LiCl: 56.9 eV^25^; LiF: 57.8 eV^24^. Cl 2*p*: LiCl: 201.5 eV^25^.


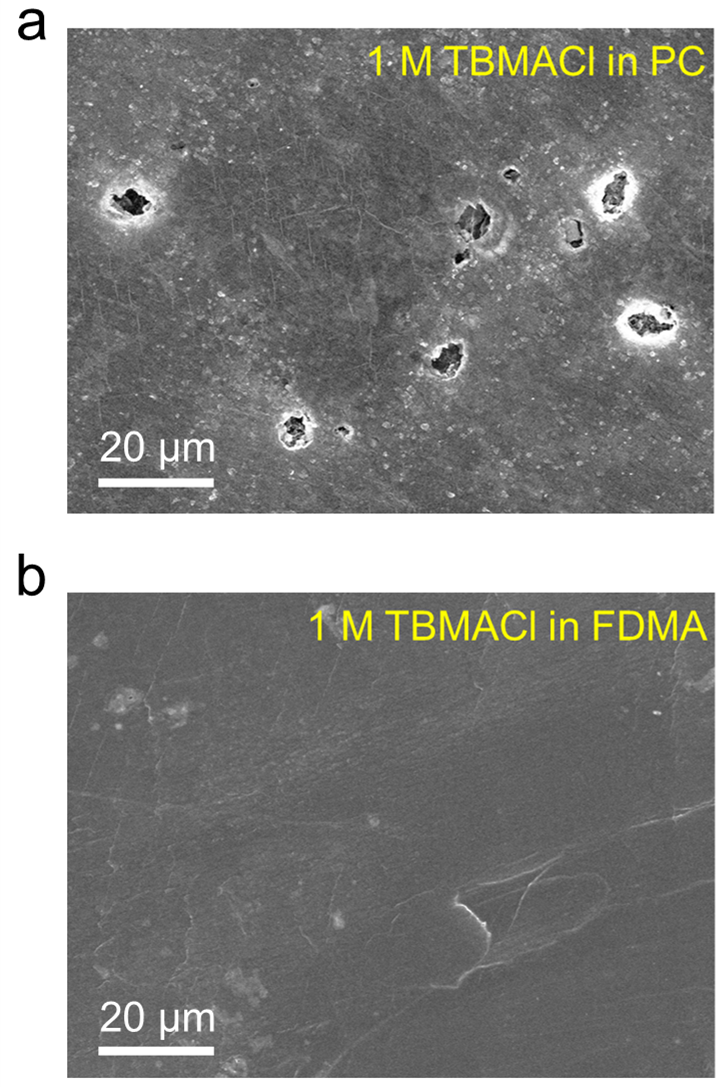


**Supplementary Figure 32** FE–SEM images of Li metal surfaces obtained from Li||FeOCl coin cells at 125 mA g^–1^ and 25 °C with **a** 1 M TBMACl in PC and **b** 1 M TBMACl in FDMA electrolytes after 3 cycles.


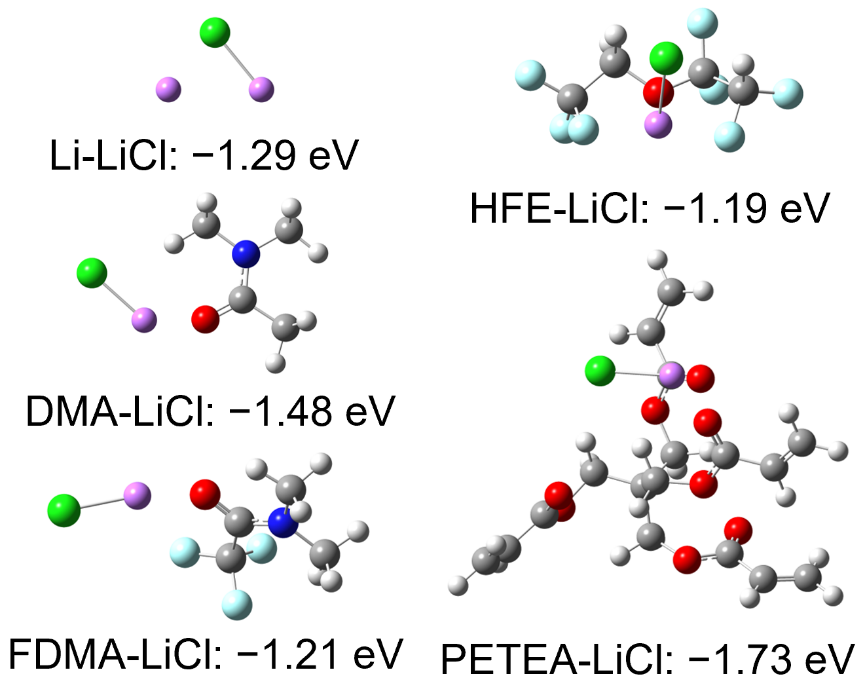


**Supplementary Figure 33** Calculated binding energies of LiCl with Li metal anode, DMA solvent, FDMA solvent, HFE solvent, and PETEA monomer. Purple, green, gray, white, red, blu,e, and cyan balls represent lithium, chlorine, carbon, hydrogen, oxygen, nitrogen, and fluorine atoms, respectively.


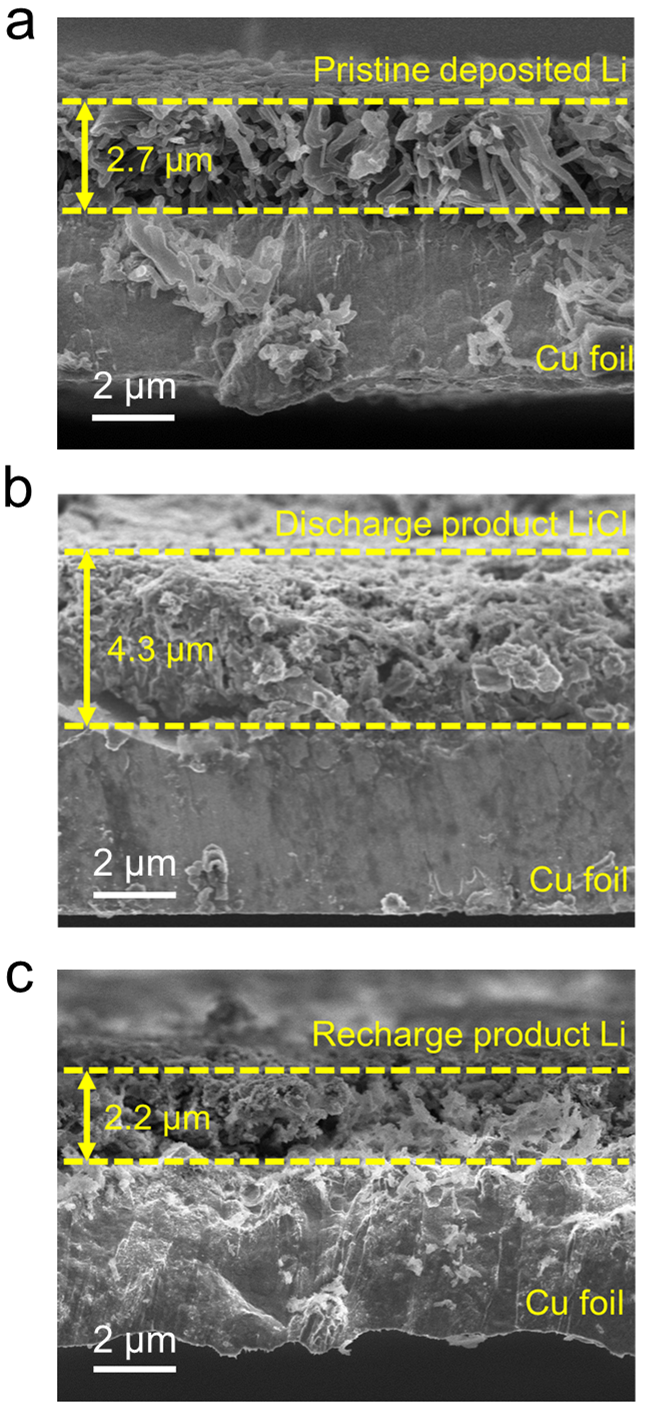


**Supplementary Figure 34** Cross–section FE–SEM images of anodes with the 4.5–μm–thick Cu foil as the current collector. **a** Pristine deposited Li metal layer obtained from a Li||Cu cell with a cut–off capacity of 1 mAh. **b** Discharge product LiCl layer and **c** recharge product Li metal layer obtained from Li|HGPE|FeOCl cells with a cut–off capacity of 1 mAh (See **Supplementary Note 7**).


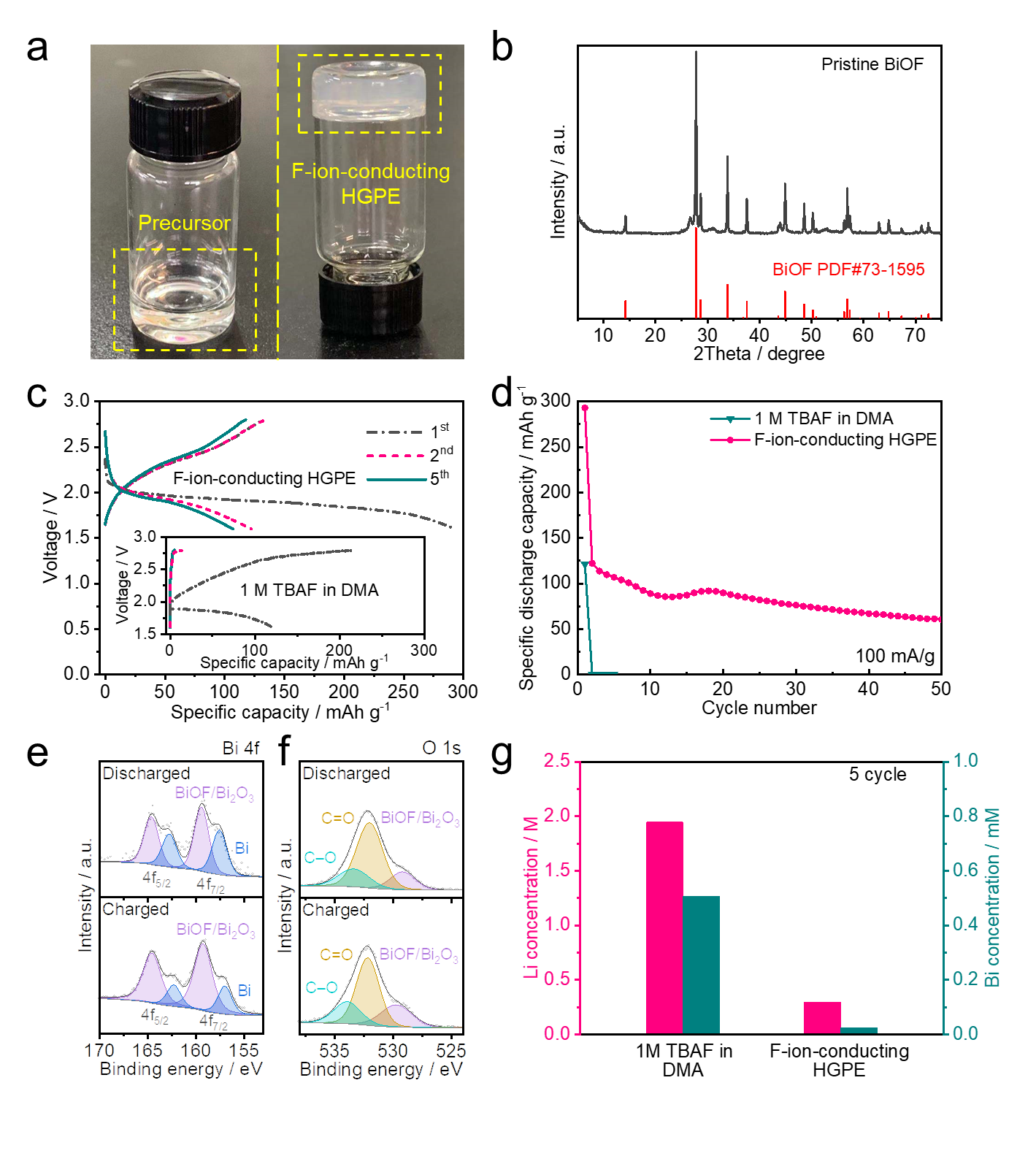


**Supplementary Figure 35** Electrochemical performance of the Li|F–ion–conducting HGPE|BiOF cell at 25 °C. **a** Optical images of precursor solution (left) and F–ion–conducting HGPE (right). **b** XRD patterns of pristine BiOF particles (in accordance with PDF#73–1595). **c** Typical galvanostatic discharge/charge profiles and **d** cycling performance of the Li||BiOF coin cells with 1 M TBAF in DMA electrolyte and F–ion–conducting HGPE between 1.6 and 2.8 V at 100 mA g^–1^. The irreversible capacity and electrochemical polarization in the initial cycle can be attributed to the grain refinement process of BiOF with large pristine particle^11, 13^. **e** Bi 4*f* and **f** O 1*s* XPS spectra of BiOF cathodes discharged/charged in F–ion–conducting HGPE. **g** Li/Bi concentrations in different electrolytes obtained from Li||BiOF cell cells after 5 cycles at 100 mA g^–1^ and 25 °C (See **Supplementary Note 9**).


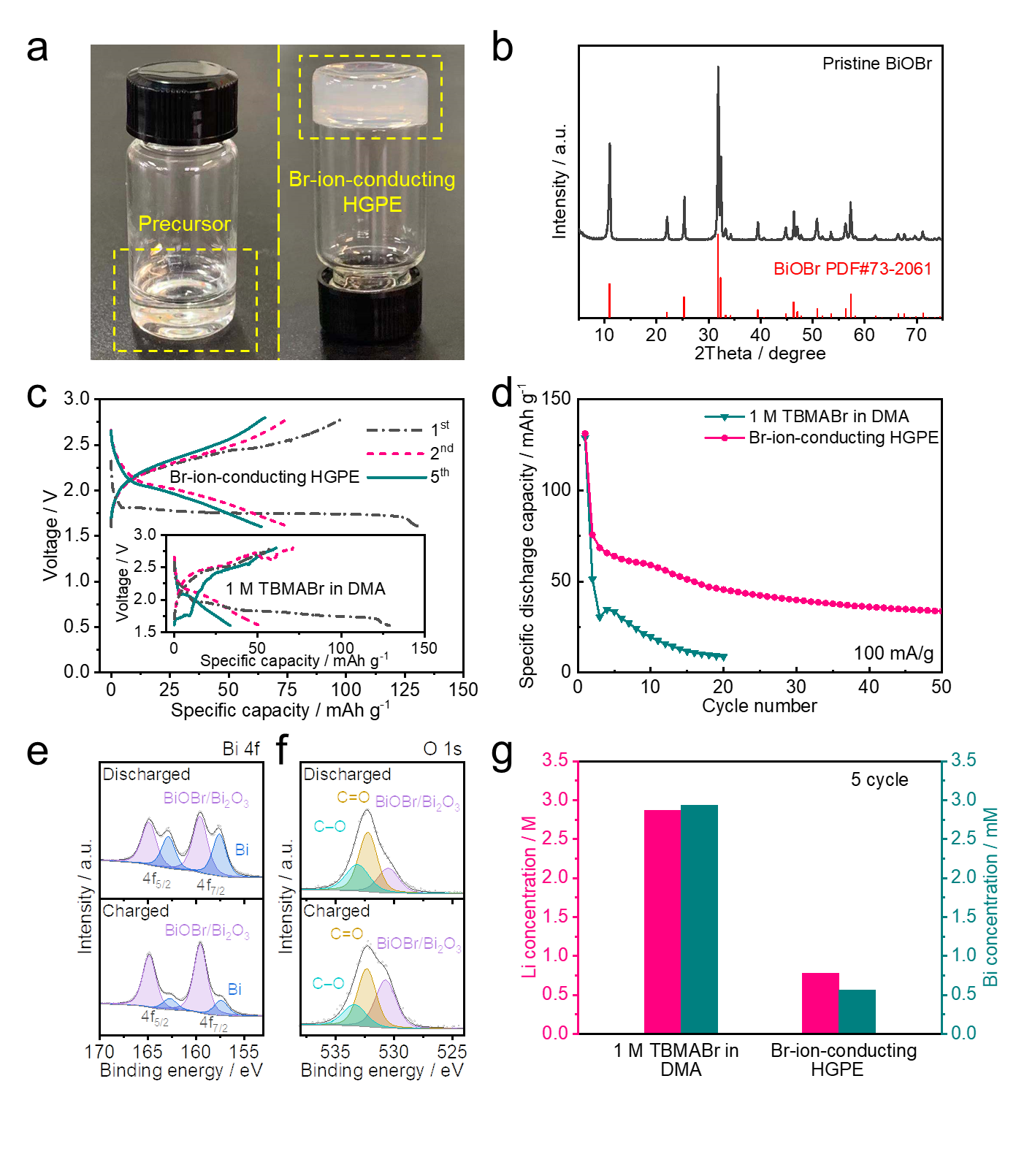


**Supplementary Figure 36** Electrochemical performance of the Li|Br–ion–conducting HGPE|BiOBr cell at 25 °C. **a** Optical images of precursor solution (left) and Br–ion–conducting HGPE (right). **b** XRD patterns of pristine BiOBr particles (in accordance with PDF#73–2061). **c** Typical galvanostatic discharge/charge profiles and **d** cycling performance of the Li||BiOBr coin cells with 1 M TBMABr in DMA electrolyte and Br–ion–conducting HGPE between 1.6 and 2.8 V at 100 mA g^–1^. The irreversible capacity and electrochemical polarization in the initial cycle can be attributed to the grain refinement process of BiOBr with large pristine particle^11, 13^. **e** Bi 4*f* and **f** O 1*s* XPS spectra of BiOBr cathodes discharged/charged in Br–ion–conducting HGPE. **g** Li/Bi concentrations in different electrolytes obtained from Li||BiOBr cell cells after 5 cycles at 100 mA g^–1^ and 25 °C (See **Supplementary Note 9**).


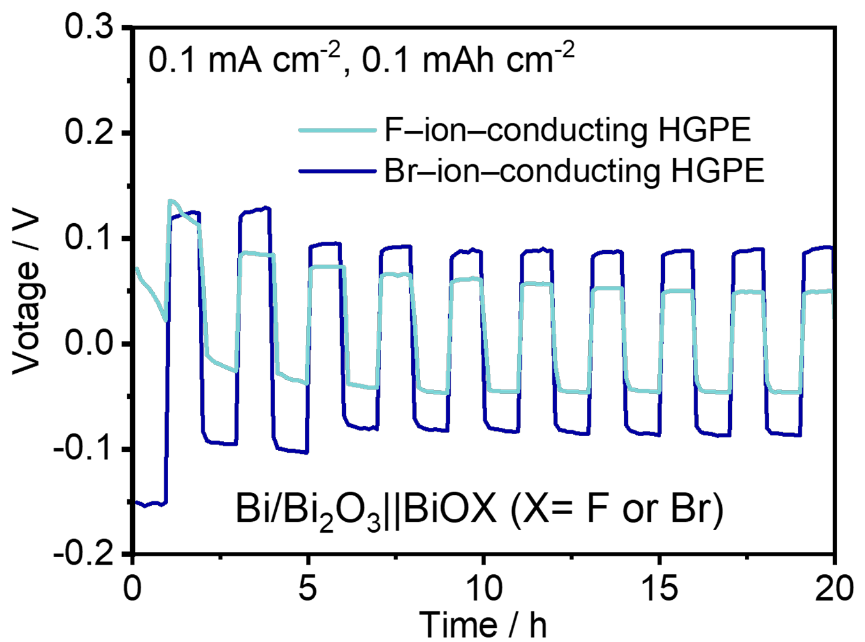


**Supplementary Figure 37.** Voltage profiles of Bi/Bi_2_O_3_|F–ion–conducting HGPE|BiOF and Bi/Bi_2_O_3_|Br–ion–conducting HGPE|BiOBr coin cells at 0.1 mA cm^–2^ and 25 °C with a cut–off the capacity of 0.1 mAh cm^–2^ (See **Supplementary Note 10**).


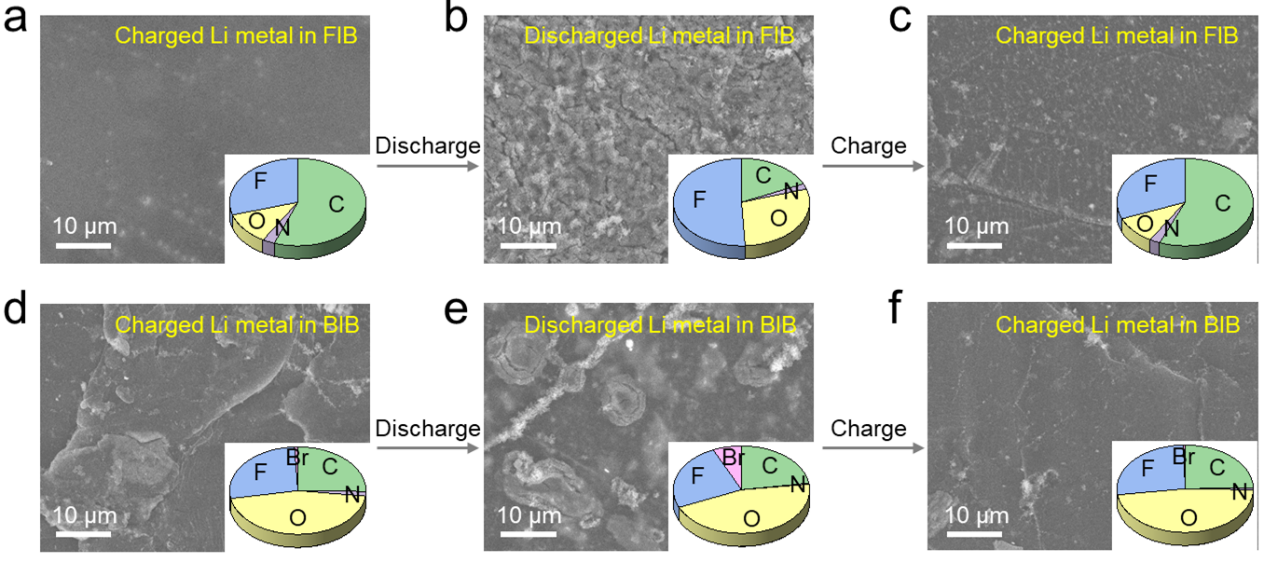


**Supplementary Figure 38**. FE–SEM images of Li metal anodes cycled in **a–c** FIBs and **d–f** BIBs full coin cells at different state of charge at at 100 mA g^–1^ and 25 °C. EDS element analyses are shown in insets. EDS element analyses are represented as atomic ratio (See **Supplementary Note 11**).

**Supplementary Tables**

**Supplementary Table 1.** Comparison of theoretical energy density and specific energy of different battery technologies in **Figure 1a**.

| Battery types | Battery Configurations | Molar Mass  (g mol^–1^) | Unit e^–^ Number n_cathode_ | Theoretical Capacity  (Ah kg^–1^) | Average Voltage (V) | Unit Cathode Mass  (g Wh^–1^) | M_anode_  (g mol^–1^) | Unit e^–^ Number n_anode_ | Unit Anode Mass  (g Wh^–1^) | Cathode Density  (g cm^–3^) | Anode Density  (g cm^–3^) | Specific Energy Density  (Wh kg^–1^) | Energy Density  (Wh L^–1^) | Ref. |
| --- | --- | --- | --- | --- | --- | --- | --- | --- | --- | --- | --- | --- | --- | --- |
| Li–ion batteries | Graphite \|\|LiFePO_4_ | 157.8 | 1 | 169.84 | 3.3 | 1.78 | 12 | 0.17 | 0.81 | 3.60 | 2.16 | 384.86 | 1147.01 | 26 |
|  | Graphite \|\| LiCoO_2_ | 97.9 | 1 | 273.75 | 3.7 | 0.99 | 12 | 0.17 | 0.73 | 5.10 | 2.16 | 583.64 | 1889.92 | 26 |
|  | Graphite \|\| LiNi_0.8_Co_0.1_Mn_0.1_O_2_ | 97.3 | 1 | 275.44 | 3.7 | 0.98 | 12 | 0.17 | 0.73 | 4.65 | 2.16 | 585.71 | 1829.64 | 26 |
| Na–ion batteries | Na \|\| NaNi_0.5_Mn_0.5_O_2_ | 111.8 | 1 | 239.71 | 3 | 1.39 | 23 | 1 | 0.29 | 5.00 | 0.97 | 596.44 | 1743.27 | 27 |
|  | Na \|\| NaNi_0.4_Cu_0.1_Mn_0.4_Ti_0.1_O_2_ | 111.6 | 1 | 240.14 | 3 | 1.39 | 23 | 1 | 0.29 | 5.00 | 0.97 | 597.33 | 1744.78 | 28 |
|  | Na \|\| Na_3_V_2_(PO_4_)_3_ | 455.8 | 2 | 117.60 | 1.7 | 5.00 | 23 | 1 | 0.50 | 5.00 | 0.97 | 181.59 | 657.05 | 29 |
| K–ion batteries | K \|\| K_0.67_Ni_0.17_Co_0.17_Mn_0.66_O_2_ | 114.5 | 0.83 | 194.27 | 3 | 1.72 | 39 | 1 | 0.49 | 5.00 | 0.86 | 454.36 | 1103.88 | 30 |
|  | K \|\| K_0.7_Fe_0.5_Mn_0.5_O_2_ | 114.8 | 1.18 | 275.47 | 2.5 | 1.45 | 39 | 1 | 0.58 | 5.00 | 0.86 | 491.61 | 1035.53 | 31 |
|  | K \|\| K_0.5_MnO_2_ | 106.5 | 0.43 | 108.21 | 2.5 | 3.70 | 39 | 1 | 0.58 | 5.00 | 0.86 | 233.71 | 706.91 | 32 |
| Fluoride–ion batteries | La \|\| CuF_2_ | 101.5 | 2 | 528.08 | 2.5 | 0.76 | 138.9 | 3 | 0.69 | 4.23 | 6.70 | 690.37 | 3543.46 | 33 |
|  | PbF_2_ \|\| LaSrMnO_4_ | 345.6 | 2 | 155.09 | 1.5 | 4.30 | 245.2 | 2 | 3.05 | 6.06 | 8.45 | 136.09 | 934.37 | 34 |
|  | Ce \|\| BiF_3_ | 266 | 3 | 302.26 | 2.5 | 1.32 | 140.1 | 3 | 0.70 | 8.30 | 6.90 | 494.95 | 3839.36 | 35 |
|  | BiF_3_ \|\| TEMPO | 172.2 | 1 | 155.63 | 0.5 | 12.85 | 266 | 3 | 6.62 | 0.86 | 8.30 | 51.37 | 63.53 | 14 |
| Chloride–ion batteries | Li \|\| FeOCl | 107.3 | 1 | 249.77 | 2.4 | 1.67 | 6.9 | 1 | 0.11 | 4.00 | 0.53 | 563.22 | 1618.26 | 11 |
|  | Li \|\| VOCl | 102.4 | 1 | 261.72 | 2.4 | 1.59 | 6.9 | 1 | 0.11 | 4.00 | 0.53 | 588.47 | 1669.72 | 36 |
|  | Li \|\| CoFe–Cl LDH | 443.4 | 2 | 120.88 | 1.7 | 4.87 | 6.9 | 1 | 0.15 | 3.50 | 0.53 | 199.30 | 597.40 | 8 |
|  | Li \|\| PPyCl | 76.8 | 0.33 | 115.16 | 2.7 | 3.22 | 6.9 | 1 | 0.10 | 0.97 | 0.53 | 301.97 | 286.18 | 16 |
|  | Ag \|\| BiOCl | 260.4 | 1 | 102.92 | 0.6 | 16.19 | 107.9 | 1 | 6.71 | 4.00 | 10.49 | 43.66 | 213.30 | 20 |

**Supplementary Table 2.** Prices of raw materials for Li– and Cl–ion batteries in **Figure 1b**. The price of the raw material is collected from the price database of Shanghai Metals Market (https://www.metal.com/price) on 01–Jun–2022.

| Raw material | Price / US$ kg^–1^ |
| --- | --- |
| Lithium hexafluorophosphate (LiPF_6_, 99.95%, battery grade) | 47.7 |
| Lithium hydroxide (LiOH, 56.5%, battery grade) | 70.5 |
| Lithium carbonate (Li_2_CO_3_, 99.5%, battery grade) | 69.3 |
| Cobalt oxide (Co_3_O_4_, Co≥72%, industrial grade) | 61.6 |
| Cobalt sulfate (CoSO_4_, Co≥20.5%, industrial grade) | 15.8 |
| Nickel sulfate (NiSO_4_, Ni≥22%, battery grade) | 8.0 |
| Tributylmethyl ammonium chloride (TBMACl, 95%, industrial grade) | 0.3 |
| Iron chloride hexahydrate (FeCl_3_·6H_2_O, 95%, industrial grade) | 0.45 |

**Supplementary Table 3.** Solubility of FeOCl and LiCl in the 0.5 M TBMACl in PP14TFSI ionic liquid electrolyte. The dissolution of FeOCl and LiCl in an ionic liquid electrolyte (*i.e.* 0.5 M TBMACl in PP_14_TFSI (1–Butyl–1–methylpiperidinium bis(trifluoromethylsulfonyl)imide))^11^ was test as follow. 0.5 mg (excess amount) of LiCl or FeOCl was immersed in such an ionic liquid electrolyte for 7 days. The supernatant was diluted by ultrapure water and measured by the ICP–OES.

| Element | Solubility |
| --- | --- |
| Fe | 2.13 mol L^–1^ |
| Li | 2.24 mmol L^–1^ |

**Supplementary Table 4.** Cl/Fe atomic ratio of FeOCl samples shown in the **Supplementary Figure 1**. The Cl/Fe atomic ratio was calculated as “Cl atomic ratio” divided by the “Fe atomic ratio” which are the statistical data obtained from the XPS survey spectra.

| FeOCl sample | Cl/Fe atomic ratio |
| --- | --- |
| Pristine | 81.8% |
| Immersed in 1 M TBMACl in PC | 13.2% |
| Self–discharged in 1 M TBMACl in HFE | 60.5% |

**Supplementary Table 5.** Bulk ionic conductivity values at 25 °C of the electrolyte samples corresponding to **Supplementary Figure 7**.

| Electrolyte sample | *σ* at 25 °C (mS cm^–1^) |
| --- | --- |
| 1 M TBMACl in PC | 5.26 |
| 1 M TBMACl in DMA | 4.48 |
| 1 M TBMACl in FDMA | 2.46 |
| 1 M TBMACl in HFE | 2.01 |
| 1 M TBMACl in FDMA: HFE | 2.76 |
| HGPE | 2.63 |

**Supplementary Table 6**. The numerical values for each equivalent circuit element and the error between the raw and fitted data shown in the **Supplementary Figure 12**. The raw EIS data was processed by Zview (Version 2.1c) software.

| Element(unit) | Value | Error |
| --- | --- | --- |
| R1 (Ω) | 30.56 | 0.86657 |
| R2 (Ω) | 30.13 | 0.95826 |
| CPE1–T (Ω^–1^ s^n^, n= CPE1–P) | 9.6652E–8 | 1.5817E–8 |
| CPE1–P (unitless) | 0.83187 | 0.014624 |
| R3 (Ω) | 128.8 | 1.2177 |
| W1–R (Ω) | 77.4 | 5.2555 |
| W1–T (s) | 0.31882 | 0.038783 |
| W1–P (unitless) | 0.30919 | 0.0022279 |
| CPE2–T (Ω^–1^ s^n^, n= CPE2–P) | 3.712E–5 | 1.165E–6 |
| CPE2–P (unitless) | 0.73274 | 0.0038687 |

**Supplementary Table 7.** Comparison of specific energy (based on the mass of cathode active material only at first cycle) and cycle life of reported CIBs using organic–solvent (OS)–based, ionic–liquid (IL)–based or aqueous (aq) electrolytes and the quasi–solid–state CIB this study (**Supplementary Figure 13**). The blanks in the table represent the missing information in the references.

| Reference  No. | Cell  configuration | Electrolyte type | Average discharge voltage (V) | Average discharge capacity  (mAh g^–1^) | Specific energy  (Wh kg^–1^) | Cycle life | Mass loading (mg cm^–2^) of the positive electrode | wt.% of active material in the positive electrode | Temperature  (°C) |
| --- | --- | --- | --- | --- | --- | --- | --- | --- | --- |
| Ref. 6 | Li\|\|VOCl  coin cell | OS | 1.5 | 123 | 184.5 | 100 | 2~3 | 56% | 25 |
| Ref. 8 | Li\|\|CoFe–Cl LDH  coin cell | OS | 1.7 | 150 | 255 | 100 | 1~1.5 | 60% | 25 |
| Ref. 1 | Li\|\|FeOCl  coin cell | OS | 2.8 | 60 | 168 | 20 |  | 60% | 50 |
| Ref. 7 | Li\|\|Sb_4_O_5_Cl_2_  coin cell | OS | 1.6 | 90 | 144 | 80 | 1.5~2 | 56% | 25 |
| Ref. 9 | Li\|\|NiMn–Cl LDH coin cell | OS | 1.7 | 140 | 238 | 150 | 1~1.5 | 70% | 25 |
| Ref. 10 | Li\|\|β–FeOOH(Cl)  coin cell | OS | 2.2 | 122 | 268.4 | 100 |  | 60% | 25 |
| Ref. 16 | Li\|\|PPyCl  coin cell | IL | 2.9 | 90 | 261 | 40 | 3.5~4.5 | 82% | 25 |
| Ref. 11 | Li\|\|FeOCl  Swagelok cell | IL | 2.3 | 60 | 138 | 30 |  | 80% | 25 |
| Ref. 12 | Mg\|\|VOCl  Swagelok cell | IL | 1.6 | 80 | 128 | 55 | 1~1.5 | 80% | 25 |
| Ref. 13 | Mg\|\|FeOCl  Swagelok cell | IL | 0.6 | 65 | 97.5 | 30 | 2.5 | 80% | 25 |
| Ref. 14 | Li\|\|FeOCl  coin cell | IL | 2.4 | 155 | 372 | 30 |  | 50% | 25 |
| Ref. 19 | Li\|\|FeOCl  coin cell | IL | 2.4 | 120 | 288 | 50 |  | 60% | 25 |
| Ref. 17 | Li\|\|PANICl_0.25_  coin cell | IL | 2.7 | 70 | 189 | 50 |  | 80% | 25 |
| Ref. 18 | Li\|\|FeOCl  coin cell | IL | 2.4 | 160 | 384 | 30 |  | 80% | 25 |
| Ref. 15 | Li\|\|FeOCl  coin cell | IL | 2.3 | 85 | 195.5 | 50 |  | 60% | 25 |
| Ref. 20 | BiOCl\|\|Ag  coin cell | aq | 0.6 | 100 | 60 | 45 |  | 50% | 25 |
| Ref. 21 | Sb_4_O_5_Cl_2_\|\|Ag  Two–electrode cell | aq | 0.7 | 37.5 | 26.25 | 50 | 1 | 70% | 25 |
| Ref. 22 | Bi\|\|AgCl  coin cell | aq | 1 | 80 | 80 | 200 |  | 60% | 25 |
| Ref. 23 | Sb\|\|AgCl  coin cell | aq | 0.95 | 65 | 61.75 | 200 |  | 70% | 25 |
| **This study** | **Li\|\|FeOCl**  **coin cell** | **OS** | **2.4** | **180** | **432** | **500** | **1~4** | **60%** | **25** |

**Supplementary Notes**

**Supplementary Note 1 Polymerization mechanism of the PETEA monomers.** As shown in **Supplementary Figure 5a**, the as–developed Cl–ion–conducting HGPE appeared as a free–standing translucent gel. FTIR of PETEA monomer and the polymer matrix of HGPE are shown in **Supplementary Figure 5b**. The PETEA exhibited absorption peaks at 1161 cm^–1^ (C–O symmetrical stretching), 1264 cm^–1^ (C–O antisymmetric stretching), 1406 and 1469 cm^–1^ (CH_2_ bending), and 1726 cm^–1^ (C=O stretching)^5^. After the heat treatment, the C=C stretching vibration peak at around 1633 cm^–1^ nearly vanished, indicating a high polymerization degree of PETEA monomer in the HGPE.

**Supplementary Note 2 Leakage tests.** During the leakage tests, 1.0 g of 1 M TBMACl in FDMA: HFE (1: 1 by volume) liquid electrolyte and 1 g precursor solution of HGPE were sealed in aluminum–plastic film pouches. After a 60 °C heat treatment to *in situ* form the HGPE, two pouches were cut by scissors and pressed under 1 kg weight for 1 min in the glovebox. By calculating the weight change of pouches, the leakage was reflected by the weight losses of 33.51 wt% and 1.81 wt% for the liquid electrolyte and HGPE, respectively. Subsequently, the two pouches were hung vertically for 5 min. The weight loss ratio was 72.18 wt% for the liquid electrolyte and 2.48 wt% for the HGPE, respectively, indicating the superior leakage resistance of the HGPE.

**Supplementary Note 3 EIS measurement analyses.** As can be seen from **Supplementary Figure 12**, the experimental and fitted spectra are quite well matched. According to the equivalent circuit in the inset of **Supplementary Figure 12**, the intersection of the impedance signal with the real axis refers to a bulk resistance (*R*_b_), reflecting the resistance of electrodes and electrolyte/separator. The depressed semicircle at high frequency can be attributed to the interphase resistance (*R*_f_) and *CPE1*, while the depressed semicircle at the medium frequency can be ascribed to the charge transfer resistance (*R*_ct_) and *CPE2*. Instead of the capacitance of the passivate interphase (*C*_f_) and double–layer capacitance (*C*_dl_), *CPE1* and *CPE2* are the constant phase elements used to take the roughness of the particle surface into account. The line at low frequency is equivalent to the Warburg impedance (*Z*_w_), which is related to the Cl ion diffusion n the porous structures of the electrodes^5^.

**Supplementary Note 4 Calculation of average voltage.** As shown in **Figure 2c**, the discharge curve was in a sloped shape rather than a typical platform shape. Therefore, it’s more precise to use integral average cell voltage as the reference of average voltage^37^. The definition of average cell voltage is as follows:

$\bar{U}=\frac{E}{Q}=\frac{\int U(t)I(t)dt}{Q}$ (1)

where $\bar{U}$ is the average discharge voltage, $E$ is the discharge energy, $Q$is the discharge capacity, $U$ is the discharge voltage, $I$ is the discharge current and $t$ is the discharge time. Further, the cell was discharged at a constant current, where $Q=\int I(t)dt=It$. The integral average voltage can be thus calculated as the following formula:

$\bar{U}=\frac{\int U(Q)dQ}{Q}$ (2)

Therefore, the average voltage is equal to the battery discharge energy (that is the integral area of the volt–capacity curve in **Figure 2c**) divided by the capacity. An average voltage of 2.4 V was accordingly obtained in **Figure 2c**.

**Supplementary Note 5 In–depth XPS spectra analyses of FeOCl–base positive electrode.** Fe 2p: FeOCl: 712.1 eV (2p_3/2_) and 724.1 eV (2p_1/2_); Fe_3_O_4_: 710.2 eV (2p_3/2_) and 723.6 eV (2p_1/2_)^2^; FeO: 708.9 eV (2p_3/2_) and 721.9 eV (2p_1/2_); elemental Fe: 707.6 eV (2p_3/2_) and 720.6 eV (2p_1/2_)^1^; Satellites peak: 718.0 eV (2p_3/2_) and 731.0 eV (2p_1/2_). O 1s: alkyl carbonate (C=O): 532.5 eV; polycarbonate (C–O): 533.5eV; Li_2_O: 529.7 eV; FeO_x_: 530.5 eV; FeOCl: 531.3 eV. Fe 3p: elemental Fe: 53.8 eV; FeO_x_: 55.9 eV; FeOCl: 57.1 eV; FeF_3_: 58.9 eV^38^. Li 1s: Li_2_O: 55.0 eV. Cl 2p: FeOCl: 199.8 eV (2p_3/2_) and 201.6 (2p_3/2_)^1^.

**Supplementary Note 6** **The analyses of the peak shift in XRD patterns of FeOCl**. It is seen that the as–prepared pristine FeOCl particles were in accordance with PDF#73–2229. The graphite paper as the current collector shows strong graphite (020) peak at 26.4°. In this work, the FeOCl cathodes were prepared by coating the NMP dispersant–based slurry on the graphite papers followed by a drying treatment. It is seen that after being fabricated as cathodes, the (010) peak of pristine FeOCl particles shifted from 11.2° to 7.5° when the cathode was not thoroughly dried. This indicates the intercalation of NMP molecules into the layered structure of FeOCl^1, 14^. Furthermore, after the FeOCl cathode was thoroughly dried at 120 °C, the FeOCl (010) peak shifted back to 11.2°, demonstrating a reversible de–intercalation of NMP molecules. After being immersed in 1 M TBMACl in PC, 1 M TBMACl in HFE, and 1 M TBMACl in DMA electrolytes and HGPE, the FeOCl (010) peak appeared at 11.8°, 11.8°, 8.7° and 6.6° in the XRD patterns of FeOCl cathodes, respectively. This difference is due to the reversible intercalation of solvent molecules into the layered structure of FeOCl.

**Supplementary Note 7** **Cross–section FE–SEM images** **analyses of negative electrode.** Based on the reaction on the anode, the reversible conversion happens between equal molars of LiCl and Li. Based on the molar weights and densities of LiCl (2.07 g cm^–3^ and 42.394 g mol^–1^, respectively) and Li (0.534 g cm^–3^ and 6.941 g mol^–1^, respectively), the molar volume of LiCl (20.48 cm^3^ mol^–1^) is about 157% larger than that of Li (13.00 cm^3^ mol^–1^), verifying a remarkable volume expansion/contraction during the charge/discharge process. This can be verified by the following experiment. First, a 2.7 μm–thick Li metal layer (1 mAh) was deposited on a 4.5 μm–thick Cu foil in a Li||Cu cell (**Supplementary Figure 34a**). The volume (thickness) variation of the anode in chloride–ion battery can be directly observed and quantitatively evaluated by using this 2.7 μm–thick Li metal rather than the 50 μm–thick Li metal. After discharged at 0.1 mA with a cut–off capacity of 1 mAh in a Li|HGPE|FeOCl cell, the pristine deposited Li layer was converted into a discharge product LiCl layer with a thickness of 4.3 μm (**Supplementary Figure 34b**), indicating a 159% volume expansion. Then, it is seen that the thickness of the recharge product Li metal layer contracted to 2.2 μm after charged with a cut–off capacity of 1 mAh. Therefore, the experiment evidence agrees with the schematic illustrations in **Figure 5c** and above theoretical prediction.

**Supplementary Note 8 Specific energy comparison.** The specific energy of our single–layered pouch cell reached up to 275.5 Wh kg^–1^ based on the electrode active materials, the actual specific energy of a practical Li||FeOCl multiple–layered full cell would be roughly estimated as 137.8 Wh kg^–1^ considering the electrode materials occupy about 50 % of the total mass in prevailing pouch cell systems^39^. This value is comparable/slightly lower than that of prevailing Na–ion full cells (*e.g.* 160 Wh kg^–1^ for the hard carbon||Prussian white system)^40^, but higher than that of Zn–ion full cells (*e.g.* 50 Wh kg^–1^ for the Zn||Al_x_V_2_O_5_ system)^41^.

**Supplementary Note 9 XPS spectra analyses and electrochemical mechanism of the FIB and BIB.** As seen from **Supplementary Figure 35e** and **Supplementary Figure 36e**, peaks at 159.5 eV (Bi 4*f*_7/2_ of bismuth oxyhalides/Bi_2_O_3_), 164.9 eV (Bi 4*f*_5/2_ of bismuth oxyhalides/Bi_2_O_3_), 157.3 eV (Bi 4*f*_7/2_ of elemental Bi), and 164.6 eV (Bi 4*f*_5/2_ of elemental Bi) appeared in the Bi 4f spectra^42, 43^. In O 1s spectra (**Supplementary Figure 35f** and **Supplementary Figure 36f**), peaks at around 530 eV were related to bismuth oxyhalides/Bi_2_O_3_^42, 43^, meanwhile, peaks at 532.5 and 533.5eV can be assigned to C=O and C–O corresponding to alkyl carbonate/polycarbonate as the oxidation products of solvents, respectively^44^. Unlike the FeOCl cathode in CIBs, the bismuth oxyhalide cathode undergoes a pathway during the discharge/charge process as follows^11, 13^:

$3 BiOX \text{+}\text{ }\text{3 e}^{\text{–}} \leftrightarrow\mathrm{Bi}_{2}O_{3} + Bi + {3 X}^{\text{–}} (X = F, Br)$ (3)

As shown in the XPS spectra of the bismuth oxyhalide cathodes (**Supplementary Figure 35e, f** and **Supplementary Figure 36e, f**), Bi_2_O_3_ and Bi metal reversibly converted to bismuth oxyhalides during cycling. These results verify the reversible conversion between bismuth oxyhalides and Bi_2_O_3_/Bi during the de–intercalation/intercalation of halide ions in the cathodes for HGPE–based FIBs and BIBs.

**Supplementary Note 10 The analyses of positive electrode in of the FIB and BIB.** To prove that the extended fluoride–ion and bromide–ion battery systems were operated based on the shuttle of fluoride–ion or bromide–ion, an experiment was designed as follows. First, the BiOX (X= F or Br) electrode was fully discharged in coin cells at 100 mA g^–1^ and 25 °C to Bi/Bi_2_O_3_ and thoroughly washed with solvents to remove the remaining electrolyte. Then, this discharged electrode was paired with a fresh BiOX (X= F or Br) electrode with the corresponding halide–ion–conducting HGPE and cycled at 0.1 mA cm^–2^ with a cut–off capacity of 0.1 mAh cm^–2^. By virtue of this method, either Li metal anode or soluble Li salt was excluded from the halide–ion battery system. As shown in **Supplementary Figure 37**, the Bi/Bi_2_O_3_||BiOX cells showed stable discharge/charge processes, indicating reversible conversions of F^–^/Br^–^ ion with BiOX electrodes based on the shuttle of halide ions.

**Supplementary Note 11 The analyses of discharge product on Li–metal negative electrode.** To verify the reversibility of halide–ion transfer on the Li metal anode during cycling, we conducted the following experiment. First, FIB (Li||BiOF cell) and BIB (Li||BiOBr cell) cells were assembled and cycled for 5 cycles to form the stable SEI on the Li anodes. Then cells were disassembled and small parts of Li metal anodes at charged state were cut off for SEM characterization. The rest of Li metal anodes were re–assembled into the halide–ion cells, and small parts from the fully discharged Li metal anodes were tested by SEM. After that, the rest of Li metal anodes were re–assembled in the halide–ion cells and re–charged for SEM characterization. As shown in **Supplementary Figure 38a–c**, in FIB, the F element ratio on the Li anode increased from 29.7 % in the charged state to 50.8 % in the discharged state, and then dropped back to 31.2 %, accompanied by a reversible change of surface roughness. The similar tendency was also observed in BIB (**Supplementary Figure 38d–f**). These results confirm a highly revisable halide–ion transfer on the anode side.

**Supplementary References**

1. Chen C., Yu T., Yang M., Zhao X. & Shen X. An All–Solid–State Rechargeable Chloride Ion Battery. *Adv. Sci.* **6**, 1802130 (2019).

2. Li J. et al. Porous Fe_2_O_3_ nanospheres anchored on activated carbon cloth for high–performance symmetric supercapacitors. *Nano Energy* **57**, 379–387 (2019).

3. Liu M. et al. Novel gel polymer electrolyte for high–performance lithium–sulfur batteries. *Nano Energy* **22**, 278–289 (2016).

4. Xu X. et al. A room–temperature sodium–sulfur battery with high capacity and stable cycling performance. *Nat. Commun.* **9**, 3870 (2018).

5. Xu X. et al. Quasi–Solid–State Dual–Ion Sodium Metal Batteries for Low–Cost Energy Storage. *Chem* **6**, 902–918 (2020).

6. Gao P. et al. VOCl as a Cathode for Rechargeable Chloride Ion Batteries. *Angew. Chem. Int. Ed.* **55**, 4285–4290 (2016).

7. Lakshmi K. P., Janas K. J. & Shaijumon M. M. Antimony oxychloride embedded graphene nanocomposite as efficient cathode material for chloride ion batteries. *J. Power Sources* **433**, 126685 (2019).

8. Yin Q. et al. CoFe–Cl Layered Double Hydroxide: A New Cathode Material for High–Performance Chloride Ion Batteries. *Adv. Funct. Mater.* **29**, 1900983 (2019).

9. Luo J. et al. NiMn–Cl Layered Double Hydroxide/Carbon Nanotube Networks for High–Performance Chloride Ion Batteries. *ACS Appl. Energ. Mater.* **3**, 4559–4568 (2020).

10. Zhao G. et al. Hollandite–type beta–FeOOH(Cl) as a new cathode material for chloride ion batteries. *Chem. Commun.* **56**, 12435–12438 (2020).

11. Zhao X., Zhao–Karger Z., Wang D. & Fichtner M. Metal oxychlorides as cathode materials for chloride ion batteries. *Angew. Chem. Int. Ed.* **52**, 13621–13624 (2013).

12. Gao P. et al. Vanadium oxychloride/magnesium electrode systems for chloride ion batteries. *ACS Appl. Mater. Interfaces* **6**, 22430–22435 (2014).

13. Zhao X. et al. Magnesium anode for chloride ion batteries. *ACS Appl. Mater. Interfaces* **6**, 10997–11000 (2014).

14. Yu T. et al. Nanoconfined Iron Oxychloride Material as a High–Performance Cathode for Rechargeable Chloride Ion Batteries. *ACS Energy Lett.* **2**, 2341–2348 (2017).

15. Yu T. T., Zhao X. Y., Ma L. Q. & Shen X. D. Intercalation and electrochemical behaviors of layered FeOCl cathode material in chloride ion battery. *Mater. Res. Bull.* **96**, 485–490 (2017).

16. Zhao X. et al. Developing Polymer Cathode Material for the Chloride Ion Battery. *ACS Appl. Mater. Interfaces* **9**, 2535–2540 (2017).

17. Zhao Z. G., Yu T. T., Miao Y. C. & Zhao X. Y. Chloride ion–doped polyaniline/carbon nanotube nanocomposite materials as new cathodes for chloride ion battery. *Electrochim. Acta* **270**, 30–36 (2018).

18. Yang R. J., Yu T. T. & Zhao X. Y. Polypyrrole–coated iron oxychloride cathode material with improved cycling stability for chloride ion batteries. *J. Alloys Compd.* **788**, 407–412 (2019).

19. Yu T. T., Yang R. J., Zhao X. Y. & Shen X. D. Polyaniline–Intercalated FeOCl Cathode Material for Chloride–Ion Batteries. *ChemElectroChem* **6**, 1761–1767 (2019).

20. Chen F. M., Leong Z. Y. & Yang H. Y. An aqueous rechargeable chloride ion battery. *Energy Stor. Mater.* **7**, 189–194 (2017).

21. Hu X. et al. Electrochemical Performance of Sb_4_O_5_Cl_2_ as a New Anode Material in Aqueous Chloride–Ion Battery. *ACS Appl. Mater. Interfaces* **11**, 9144–9148 (2019).

22. Zhang Z. et al. The composite electrode of Bi@carbon–texture derived from metal–organic frameworks for aqueous chloride ion battery. *Ionics* **26**, 2395–2403 (2019).

23. Zhang Q. et al. Sb nanoparticle decorated rGO as a new anode material in aqueous chloride ion batteries. *Nanoscale* **12**, 12268–12274 (2020).

24. Wang Q. et al. Interface chemistry of an amide electrolyte for highly reversible lithium metal batteries. *Nat. Commun.* **11**, 4188 (2020).

25. Auvergniot J. et al. Redox activity of argyrodite Li_6_PS_5_Cl electrolyte in all–solid–state Li–ion battery: An XPS study. *Solid State Ion.* **300**, 78–85 (2017).

26. Wu F. & Yushin G. Conversion cathodes for rechargeable lithium and lithium–ion batteries. *Energy Environ. Sci.* **10**, 435–459 (2017).

27. Wang P.–F., You Y., Yin Y.–X. & Guo Y.–G. An O3–type NaNi_0.5_Mn_0.5_O_2_cathode for sodium–ion batteries with improved rate performance and cycling stability. *J. Mater. Chem. A* **4**, 17660–17664 (2016).

28. Wang Q. et al. Reaching the Energy Density Limit of Layered O3‐NaNi_0.5_Mn_0.5_O_2_ Electrodes via Dual Cu and Ti Substitution. *Adv. Energy Mater.* **9**, 1901785 (2019).

29. Jiang Y. et al. Highly Reversible Na Storage in Na_3_V_2_(PO_4_)_3_ by Optimizing Nanostructure and Rational Surface Engineering. *Adv. Energy Mater.* **8**, (2018).

30. Liu C. et al. K_0.67_Ni_0.17_Co_0.17_Mn_0.66_O_2_: A cathode material for potassium–ion battery. *Electrochem. Commun.* **82**, 150–154 (2017).

31. Kim H. et al. Investigation of Potassium Storage in Layered P3–Type K_0.5_MnO_2_ Cathode. *Adv. Mater.* **29**, 1702480 (2017).

32. Wang X. et al. New–type K_0.7_Fe_0.5_Mn_0.5_O_2_ cathode with an expanded and stabilized interlayer structure for high–capacity sodium–ion batteries. *Nano Energy* **35**, 71–78 (2017).

33. Thieu D. T. et al. CuF_2_ as Reversible Cathode for Fluoride Ion Batteries. *Adv. Funct. Mater.* **27**, 1701051 (2017).

34. Nowroozi M. A., Wissel K., Rohrer J., Munnangi A. R. & Clemens O. LaSrMnO_4_: Reversible Electrochemical Intercalation of Fluoride Ions in the Context of Fluoride Ion Batteries. *Chem. Mater.* **29**, 3441–3453 (2017).

35. Bhatia H. et al. Conductivity Optimization of Tysonite–type La_1–x_Ba_x_F_3–x_ Solid Electrolytes for Advanced Fluoride Ion Battery. *ACS Appl. Mater. Interfaces* **9**, 23707–23715 (2017).

36. Hou X. et al. An Aqueous Rechargeable Fluoride Ion Battery with Dual Fluoride Electrodes. *J. Electrochem. Soc.* **166**, A2419–A2424 (2019).

37. Balaish M., Kraytsberg A. & Ein–Eli Y. Distinct view on batteries performance analysis. *J. Electroanal. Chem.* **707**, 85–88 (2013).

38. Zhou H. et al. Controlled formation of mixed nanoscale domains of high capacity Fe_2_O_3_–FeF_3_ conversion compounds by direct fluorination. *ACS Nano* **9**, 2530–2539 (2015).

39. Zhou D. et al. A Novel Lithiated Silicon–Sulfur Battery Exploiting an Optimized Solid–Like Electrolyte to Enhance Safety and Cycle Life. *Small* **13**, 1602015 (2017).

40. Time for lithium–ion alternatives. *Nat. Energy* **7**, 461–461 (2022).

41. Wan F. et al. A Universal Compensation Strategy to Anchor Polar Organic Molecules in Bilayered Hydrated Vanadates for Promoting Aqueous Zinc–Ion Storage. *Adv. Mater.* **33**, e2102701 (2021).

42. Qiang Z., Zhu S., Li T. & Li F. Excellent photo– and sono– catalytic BiOF/Bi_2_O_3_ heterojunction nanoflakes synthesized via pH–dependent and ionic liquid assisted solvothermal method. *Mater. Today Commun.* **23**, 100980 (2020).

43. Yu H. et al. Liquid–Phase Exfoliation into Monolayered BiOBr Nanosheets for Photocatalytic Oxidation and Reduction. *ACS Sustain. Chem. Eng.* **5**, 10499–10508 (2017).

44. Jaumaux P. et al. Localized Water–In–Salt Electrolyte for Aqueous Lithium–Ion Batteries. *Angew. Chem. Int. Ed.* **60**, 19965–19973 (2021).
