# Supplementary material for: Electrolyte design principles for developing quasi-solid-state rechargeable halide-ion batteries
Source: Nat Commun. 2023 Feb 18;14:925. doi: 10.1038/s41467-023-36622-w (PMC9938900; doi:10.1038/s41467-023-36622-w)
Supplement: Supplementary file 3 — Description of Additional Supplementary Files [file 41467_2023_36622_MOESM3_ESM.pdf]

Description of Additional Supplementary Files

File Name: Supplementary Movie 1

Description: The ignition test of 1 M TBMACl in DMA electrolyte. The self–extinguishing time (SET) of 1 M TBMACl in DMA electrolyte is 50 s g^{-1}.

File Name: Supplementary Movie 2

Description: The ignition test of HGPE. The self–extinguishing time (SET) of HGPE electrolyte is 0 s g^{-1}.

File Name: Supplementary Movie 3

Description: The LED lighting test of Li|1 M TBMACl in DMA|FeOCl pouch cell before and after severe shape deformation.

File Name: Supplementary Movie 4

Description: The LED lighting test of Li|HGPE|FeOCl pouch cell before and after severe shape deformation.
